# Design, synthesis, and mechanistic evaluation of propargylated salicylaldehyde derivatives as dual apoptosis–autophagy modulator for pancreatic cancer

**DOI:** 10.1007/s00210-026-05127-w

**Published:** 2026-02-24

**Authors:** Filiz Taspinar, Karina Amudi, Nurettin Menges

**Affiliations:** 1 Department of Physiology, Faculty of Medicine, Aksaray University, Aksaray, 68300 Türkiye; 2 Science and Technology Research and Application Center (BITAM), Necmettin Erbakan University, Konya, 42090 Türkiye; 3 Biomedical Engineering, Faculty of Engineering, Necmettin Erbakan University, Konya, 42090 Türkiye

**Keywords:** Apoptosis, Autophagy, Gene expression, Substitution, Spectral analysis

## Abstract

In this study, eight novel propargylated salicylaldehyde derivatives and one propargylated indole derivative were successfully synthesized and evaluated for their cytotoxic activities against the human pancreatic cancer cell line PANC-1. Among the nine tested molecules, molecules 4 and 6 exhibited the most pronounced cytotoxic effects, with IC_50_ values of 65.7 and 80.6 µM, respectively. Additionally, cytotoxicities of molecule 4 and 6 were also tested against prostate cancer (PC-3), lung adenocarcinoma (A549), and human dermal fibroblasts (hDF). Even though molecule 4 exhibited more moderate effects, gene expression analysis revealed that molecule 6 was associated with increased transcriptional expression of pro-apoptotic markers (Bax, Bad, Casp8, Casp9) and autophagy-associated genes (ATG6, ATG7, ATG8, AMBRA1), suggesting transcriptional engagement of apoptosis- and autophagy-related regulatory networks. These results were corroborated by complementary in silico docking studies, which demonstrated that molecule 6 had favorable binding interactions with the active sites of AMBRA1, BAX, ATG8, and Caspase-8. Despite having IC_50_ values in the micromolar range, molecule 6 shows promise as an early-stage hit molecule for further optimization and preclinical investigation in pancreatic cancer models. In addition to all important results, *in ovo* HET-CAM analysis also revealed that both molecule 4 and molecule 6 were non-irritant, as no hemorrhage, vascular lysis, or coagulation was observed, resulting in an irritation score of zero. This favorable tolerability profile indicates the absence of acute membrane toxicity and supports the suitability of these molecules for further biological evaluation.

**Supplementary information:**

The online version contains supplementary material available at 10.1007/s00210-026-05127-w.

## Introduction

Pancreatic cancer remains one of the most aggressive and lethal malignancies worldwide, ranking as the seventh leading cause of cancer-related mortality. Despite advances in diagnostic and therapeutic modalities, the prognosis for patients with pancreatic cancer continues to be dismal, with a five-year survival rate persistently below 10% (Wang et al. 2020). This poor outcome is primarily attributable to its asymptomatic nature during the early stages and the lack of effective biomarkers, which often results in diagnosis at advanced, inoperable stages. Moreover, pancreatic tumors are notably refractory to conventional treatment modalities such as chemotherapy and radiotherapy, necessitating the exploration of alternative therapeutic strategies and molecular targets.

In recent years, there has been a growing focus on deciphering the intricate cellular and molecular mechanisms that underlie pancreatic tumorigenesis and therapy resistance. Among these, autophagy and apoptosis have emerged as two critical and interrelated pathways that play pivotal roles in cancer cell fate determination. Autophagy, a tightly regulated catabolic process, is responsible for the degradation and recycling of intracellular organelles and proteins, thereby maintaining cellular homeostasis under both basal and stress conditions (Zhang et al. 2024). While autophagy functions as a tumor-suppressive mechanism in the early stages of carcinogenesis by mitigating oxidative stress and genomic instability, it can paradoxically support tumor cell survival in advanced cancers by enabling adaptation to hypoxic and nutrient-deprived microenvironments (Fang et al. 2022). In pancreatic cancer, elevated basal autophagic activity has been identified as a hallmark feature, contributing to tumor maintenance, metabolic reprogramming, and resistance to cytotoxic agents (Piffoux et al. 2021). However, emerging evidence suggests that overstimulation or dysregulation of the autophagy machinery may trigger autophagic cell death, offering a novel avenue for therapeutic intervention.

Simultaneously, apoptosis—a form of programmed cell death—remains a cornerstone mechanism in cancer suppression and a key target for anticancer therapy. The evasion of apoptosis is a defining characteristic of malignancies and a major contributor to therapeutic resistance. The crosstalk between autophagy and apoptosis adds another layer of complexity, as these pathways can act synergistically or antagonistically depending on cellular context, stimulus type, and intensity. Understanding this interplay is critical for the rational design of agents capable of modulating both pathways to overcome resistance mechanisms in pancreatic cancer.

In the realm of anticancer drug discovery, small molecules that selectively influence apoptotic and autophagic signaling have shown considerable promise (Khan et al. 2022; Song et al. 2023; Liao et al.^2016^; Ranjan and Srivastava 2016; Xia et al. 2009; Patel et al. 2023; Güçlü et al. 2018). Numerous substances (such as Vorinostat) alter both pathways, but frequently in an indirect way, with only preclinical data, poor selectivity, or systemic toxicity. PDAC-specific small molecules that specifically regulate both autophagy and apoptosis are comparatively rare. (Chiao et al. 2013).

Among these, propargylated molecules have gained attention due to their potential to covalently modify nucleophilic residues on target proteins, often leading to durable biological effects (Kaur et al. 2021, Hofmanova et al. 2023, Weiss et al. 2015). Structurally, the incorporation of a propargyl moiety not only enhances molecular rigidity and metabolic stability but also facilitates unique interactions with enzymatic and non-enzymatic targets. Of particular interest are salicylaldehyde derivatives, which exhibit broad-spectrum biological activities, including the induction of oxidative stress, DNA damage, modulation of cellular signaling pathways, inhibition of key enzymes, and even anti-mycotoxigenic and covalent lysine-targeting activities (Zhang et al. 2022; Ji et al. 2018; Yang et al. 2022; Singh et al. 2024; Kim et al. 2024; Mason et al. 2024; Fan et al. 2010; Fei et al. 2025). These multifaceted properties make them attractive scaffolds for the development of novel anticancer agents.

Given the urgent clinical need for innovative therapies against pancreatic cancer, our study aims to explore the therapeutic potential of propargylated salicylaldehyde and indole derivatives. We hypothesize that these molecules can exert potent cytotoxic effects through simultaneous activation of apoptotic and autophagic mechanisms. Through rational design, synthesis, and biological evaluation, we seek to identify candidate molecules capable of inducing selective cytotoxicity in pancreatic cancer cells, while providing mechanistic insights into their mode of action. The outcomes of this study may contribute to the development of new chemical entities for targeted pancreatic cancer therapy.

Multi-pathway cell-death engagement is increasingly explored to overcome adaptive survival mechanisms because pancreatic cancer remains highly therapy-resistant. While apoptosis induction has long been pursued, autophagy can function as a context-dependent survival program and contribute to drug resistance in pancreatic cancer models. Recent work also underscores the value of combination strategies to prevent resistance—for example, one study reported durable regression of orthotopic pancreatic tumors with no evidence of resistance using a targeted triple-combination approach against KRAS/EGFR/STAT3 signaling (Liaki et al. 2025). However, despite these advances, direct and systematic evidence that dual apoptosis–autophagy targeting has been established as a defined therapeutic strategy in pancreatic cancer models remains limited and fragmented, with outcomes varying by model and by whether autophagy is cytoprotective or cytotoxic—thereby defining the key research gap addressed in this study.

## Result and discussion

### Synthesis of the molecules

**Scheme 1.**
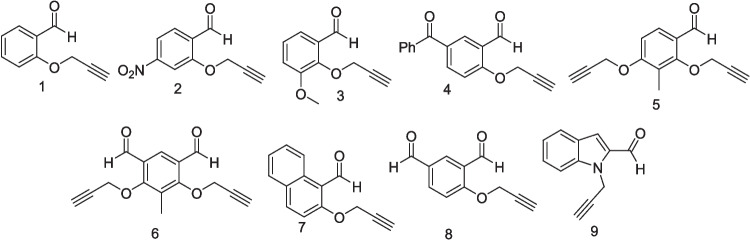
Propargylated of salicyl aldehydes and NH of indole ring

We used some commercially available salicylaldehyde derivatives for propargylation, while others were synthesized in our research laboratory (Uygun et al.^2022^). The detailed reaction scheme for this process is provided below. The formylation method was used to synthesize salicylaldehyde derivatives. For this purpose, salicylaldehyde derivatives were obtained by using HMTA in an acidic medium (TL et al. 2025) Finally, eight different salicylaldehydes were utilized for propargylation reaction (Scheme 1). In addition to salicylaldehyde derivatives, the NH group of the indole ring was also propargylated to contribute to the structure–activity relationship.

**Fig. 1:**
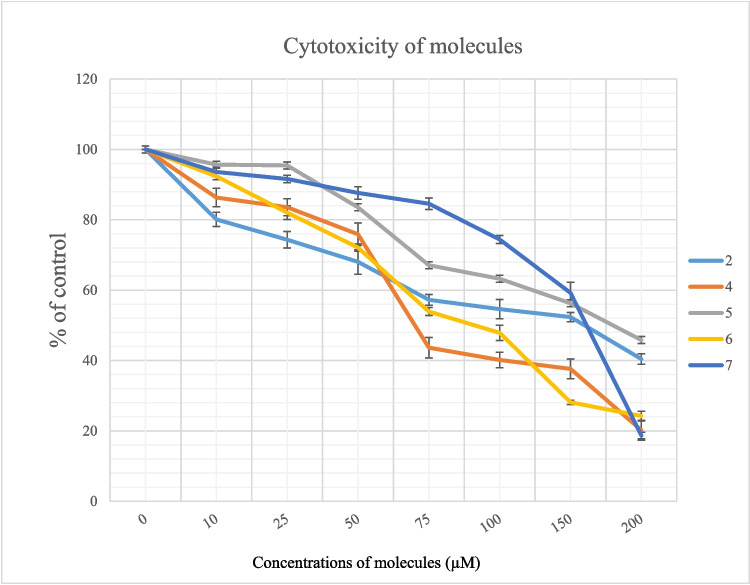
Dose-dependent cytotoxic effects of synthesized molecules **2, 4, 5, 6** and **7** on PANC-1 cell line

**Fig. 2:**
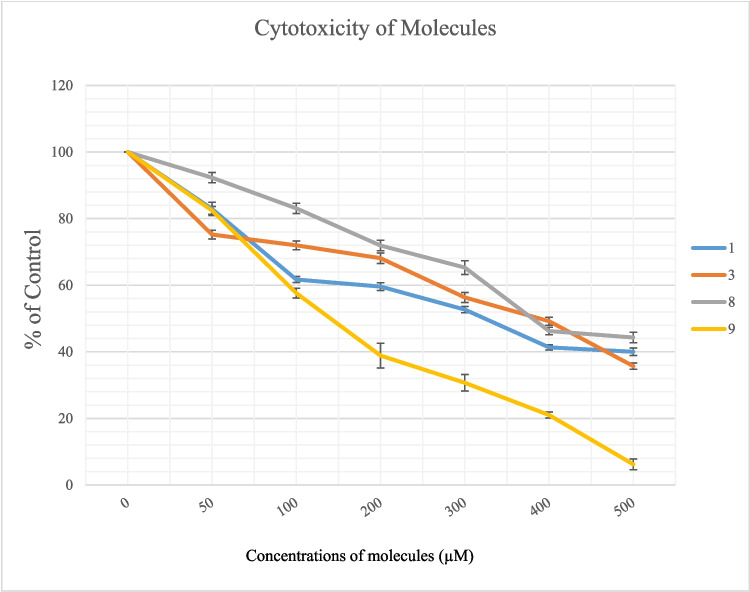
Dose-dependent cytotoxic effects of synthesized molecules **1, 3, 8,** and **9** on PANC-1 cell line

A series of eight propargylated salicylaldehyde and one propargylated indole aldehyde derivatives were synthesized and evaluated for their cytotoxic activity against the PANC-1 pancreatic cancer cell line (Figs. 1 and 2, Table 1). The IC_50_ values obtained from these assays provide insights into the structure–activity relationship (SAR) of these molecules, revealing the influence of specific substituents and structural variations on their biological activity.

**Table 1:** IC_50_ values (µM) of the tested molecules on PANC-1 cells after 72 h of treatment

Molecules	IC_50_ (µM ± SD, 72 h)
1	323.4 ± 0.9
2	161.1 ± 1.9
3	365.8 ± 1.1
4	65.7 ± 2.4
5	183.0 ± 1.4
6	80.6 ± 1.2
7	153.2 ± 1.4
8	381.0 ± 1.3
9	163.9 ± 1.6

PANC-1 is widely recognized as a highly chemoresistant pancreatic cancer line, often displaying limited responses to many standard drugs. This characteristic may contribute to the high IC₅₀ values observed in our study. To place these results into context, we also evaluated Molecules 4 and 6 in three additional cell lines (PC-3 (human prostat cancer cell line, A549 (lung adenocarcinoma), and hDF (dermal fibroblast)). The IC₅₀ values varied across cell types, reflecting cell-line–specific sensitivity: for example, Molecule 4 showed lower IC_50_ values in PC-3 (51.7 ± 2.2 µM) and hDF (56.1 ± 2.3 µM), whereas A549 exhibited considerably higher IC_50_ values (180.8 ± 2.3 µM). These comparisons indicate that PANC-1 is not universally the most resistant model but remains among the more difficult-to-treat lines, consistent with its well-documented chemoresistant phenotype (Table 2).

**Table 2:** The IC_50_ values (μM) of Molecules 4 and 6 against different cell lines after 72 h incubation

Cell Line	IC_50_ (µM ± SD, 72 h) Molecule 4	IC_50_ (µM ± SD, 72 h) Molecule 6
PANC-1	65.7 ± 2.4	80.6 ± 1.2
PC-3	51.7 ± 2.2	103.9 ± 2.3
A549	180.8 ± 2.3	206.6 ± 2.4
hDF	56.1 ± 2.3	88.4 ± 2.4

Based on the IC_50_ values obtained across cancer cell lines and the hDF normal cell line, both molecules display relatively low therapeutic indices. The comparable or higher cytotoxicity observed in hDF cells indicates insufficient selectivity toward cancer cells, limiting their potential as safe therapeutic candidates. This issue will be focused in future studies.

The cytotoxic profiles of Molecule 4 and Molecule 6 were interpreted with reference to unpublished internal data showing that 5-fluorouracil (5-FU) exhibits a 72-h IC_50_ value of approximately 20.2 µM in PANC-1 cells. Although Molecule 4 and Molecule 6 display higher IC_50_ values than 5-FU, both molecules demonstrate measurable and biologically relevant cytotoxic activity, indicating that they may serve as preliminary hit candidates suitable for further optimization and mechanistic investigation.

The observed range of cytotoxicities for molecules 4 and 6 is not unexpected during early hit identification, particularly in PANC-1 cells, which are well known for their strong drug resistance and limited responsiveness to many standard chemotherapeutics. Furthermore, because the MTT assay measures mitochondrial metabolic activity rather than direct cell death, the apparent cytotoxicity may be underestimated compared with orthogonal viability or apoptosis assays.

### Structure–activity relationships for synthesized molecules

Molecules **4** (65.7 µM) in which the phenyl substituent on the salicylaldehyde scaffold appears to enhance the cytotoxic activity significantly, likely due to increased hydrophobic interactions or π-π stacking with cellular targets. In molecule **6** (80.6 µM), the extended conjugation and presence of multiple aldehyde groups may improve molecular interactions with the target, contributing to its higher activity. On the other hand, while molecules **2** (161.1 µM), **5** (183 µM), **7** (153.2 µM), and **9** (163.9 µM) exhibit intermediate activity, molecules **1** (323.4 µM), **3** (365.8 µM), and **8** (381.0 µM) did not show promising cytotoxic effect. The presence of electron-donating or withdrawing groups at specific positions affects their binding efficiency. Molecule **9**, with an indole NH group propargylated, demonstrates a relatively stronger activity compared to other derivatives in this category, indicating that indole-based modifications enhance binding potential when molecule **1** (323.3 µM) was taken into account. It is worthy to say that molecules **3** (365.8 µM) and **8** (380.9 µM) which have some functionality, did not give good results. These three molecules show lower activity, potentially due to the lack of substituents or structural features that facilitate binding. For instance, molecule **3** with an OCH_3_ group and molecule **8** with minimal modifications do not contribute significantly to target interactions (Bildirici et al. 2018; Kuzu et al. 2025).

**Fig. 3:**
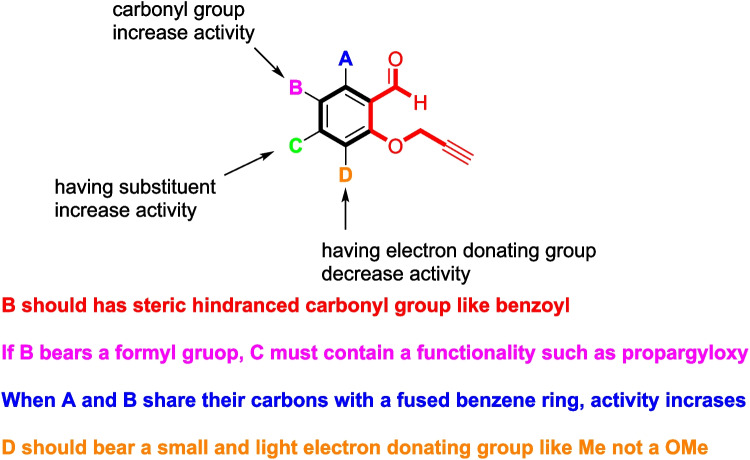
Structure–activity relationship for synthesized molecules

After SAR investigation, some points should be highlights (Fig. 3);

- **(i)** Substituent Effects

The introduction of hydrophobic (e.g., benzoyl in molecule **4**) or electron-withdrawing group (nitro in molecule **2**) improves activity by enhancing molecular interactions with cellular targets.

- **(ii)** Scaffold Optimization

Molecules with extended conjugation (e.g., molecule **6)** exhibit higher activity, suggesting that electronic and structural features might play a critical role.

- **(iii)** Propargylation of Indole

The NH group modification in the indole ring of molecule **9** contributes positively to activity, supporting the hypothesis that heterocyclic core modifications might enhance target engagement.

Molecule 4’s increased activity indicates that hydrophobic benzoyl substitution boosts target interaction, most likely via π–π stacking. Increased molecular rigidity and electron delocalization benefit molecule 6, which has two propargylated aldehyde functions and may enhance binding to autophagic and apoptotic proteins. On the other hand, electron-donating groups like methoxy (molecule 3) seem to decrease affinity, maybe as a result of a compromised electrophilic interaction profile needed for target engagement.

The study highlights the importance of structural modifications in determining the cytotoxic potential of propargylated salicylaldehyde derivatives. Molecules **4** and **6** emerged as the most promising candidates, demonstrating the highest activity against the PANC-1 cell line. For this reason, we have studied some important apoptotic and autophagic genes to understand the mechanism of these two potent molecules.

### Investigation on molecular pathways

**Fig. 4:**
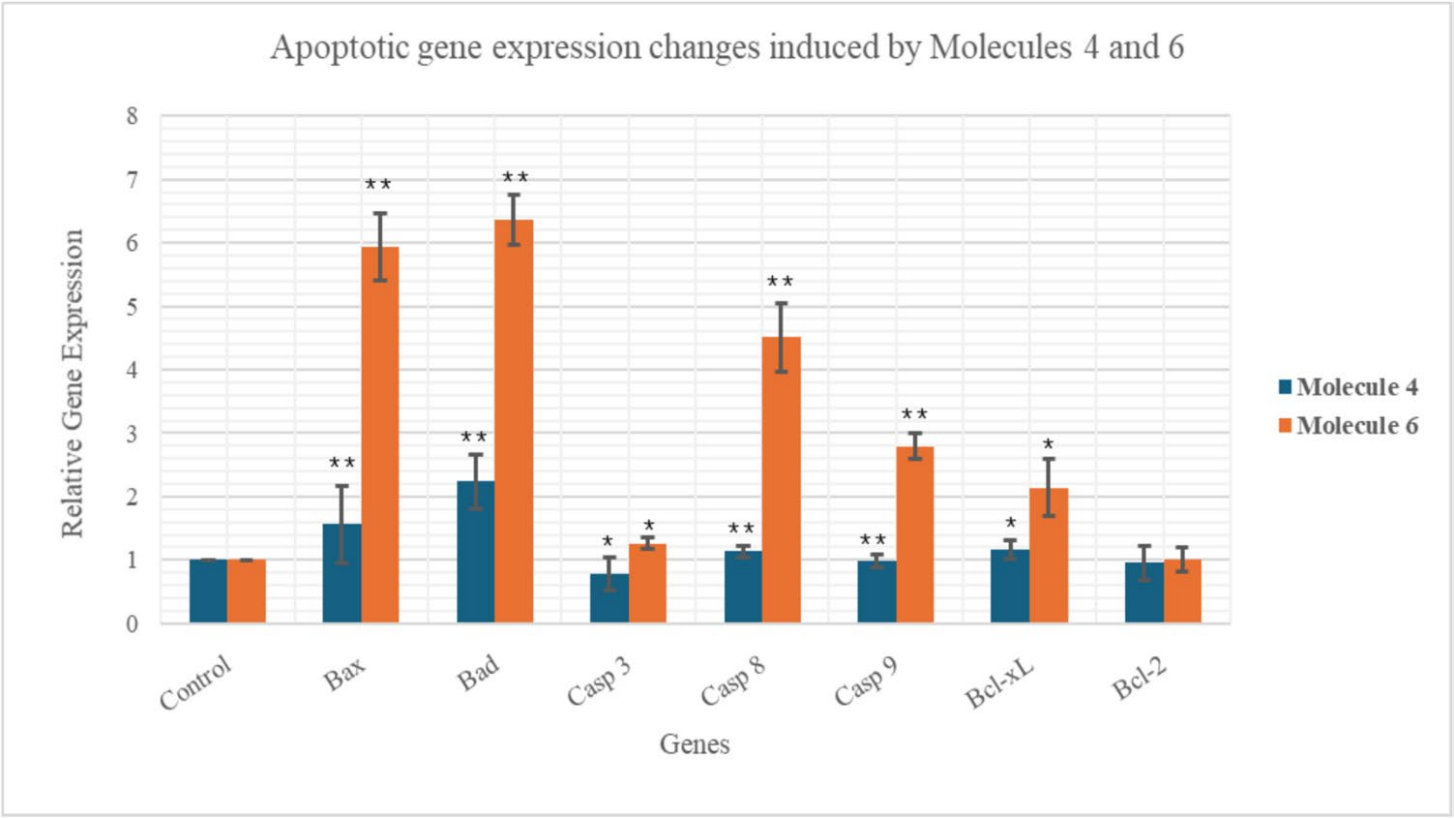
Alterations in apoptotic gene expression profiles following treatment with Molecules 4 and 6 in PANC-1 cells. Data are presented as mean ± SD from biological replicates (*n* = 3), with each reaction performed in triplicate. Statistical significance: *p < 0.01, **p < 0.001 compared with the untreated control group

**Fig. 5:**
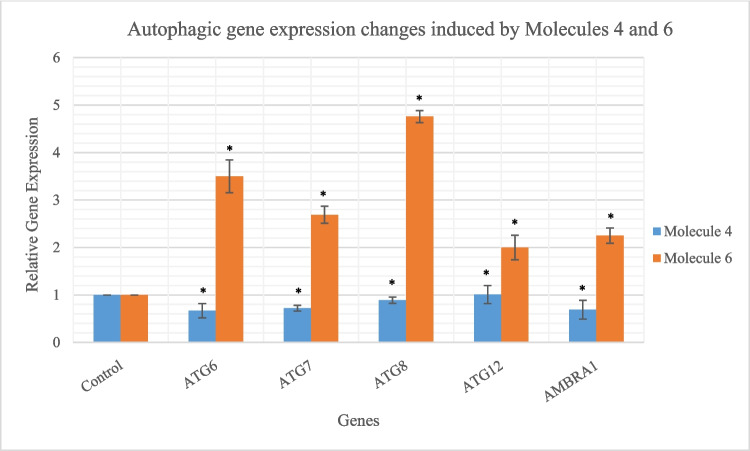
Alterations in autophagy-related gene expression profiles following treatment with Molecules 4 and 6 in PANC-1 cells. Data are presented as mean ± SD from biological replicates (*n* = 3), with each reaction performed in triplicate. Statistical significance: *p < 0.001 compared with the untreated control group

Molecules 4 and 6, identified as the most active propargylated salicylaldehyde derivatives, showed marked cytotoxic effects in PANC-1 cells. To clarify their molecular actions, we evaluated key apoptotic and autophagy-related genes (Figs. 4 and 5). Gene expression levels for apoptotic markers (Casp3, Casp8, Casp9, Bax, Bad, Bcl-2, Bcl-XL) and autophagy-related markers (ATG6, ATG7, ATG8, ATG12, AMBRA1) were quantified and normalized to untreated controls.

#### Apoptosis pathway modulation

Apoptosis is a programmed cell-death mechanism that plays a critical role in eliminating damaged or abnormal cells (Turabekova et al. 2011). Molecules 4 and 6 modulated several apoptosis-related genes, with molecule 6 showing stronger activity (Fig. 4).

## Upregulation of bax and bad (pro-apoptotic genes)

Molecule **6** dramatically increased Bax expression by 5.93-fold, suggesting a potent activation of the intrinsic apoptotic pathway (Li et al. 2025). This significant upregulation is indicative of the molecule’s ability to disrupt mitochondrial integrity and trigger cell death. Molecule **4** also showed an increase in Bax levels (1.56-fold). Molecule 4 induced a statistically significant upregulation of Bad gene expression (2.24-fold, *p* < 0.001) compared to the control group, whereas this increase was more pronounced in molecule **6** (6.36-fold, *p* < 0.001).

## Upregulation of BcI-XL and Bcl −2(Anti-apoptotic Genes)

Molecule **6** increased BcI-X_L_ expression (2.62-fold), while molecule **4** exhibited a modest increase (1.3-fold) (Riahi et al. 2008; Murphy et al. 2000). However, the elevated Bax/BcI-X_L_ ratio for molecule **6** suggests that the apoptotic signals outweigh the anti-apoptotic mechanisms, favoring cell death. Also, no statistically significant alteration in Bcl-2 mRNA levels was observed relative to controls, which is consistent with the triggering of the apoptotic pathway.

## Caspase activation

Molecule **6** strongly upregulated the expression of Casp8 (4.86-fold) and Casp9 (2.63-fold), indicating the activation of both the extrinsic and intrinsic apoptotic pathways (Akgül et al. 2020). Molecule **6**’s dual activation of these pathways underscores its robust pro-apoptotic effects.

In contrast, molecule **4** showed a milder upregulation of Casp8 (1.23-fold) and a slight decrease in Casp9 levels (0.92-fold), indicating a preference for extrinsic apoptosis. Interestingly, Casp3, the effector caspase, was moderately upregulated by molecule **6** (1.26-fold), while molecule **4** reduced its expression (0.64-fold), suggesting a more limited activation of the effector phase.

### Autophagy pathway modulation

Autophagy plays a dual role in cancer biology, functioning both as a survival mechanism and a potential route to cell death under specific conditions (Akkoc et al. 2023).

Molecule 6 treatment was associated with increased mRNA expression levels of several autophagy-related genes, including ATG6 (3.1-fold), ATG7 (2.87-fold), ATG8 (4.87-fold), and ATG12 (2.23-fold), relative to control conditions. These genes are known to participate in regulatory processes associated with autophagosome formation and autophagy-related signaling pathways (Collier et al. 2021; Deniz et al. 2022). The relatively higher transcriptional level of ATG8, which is commonly used as an autophagy-associated marker, was also observed following molecule 6 treatment.

These transcriptional changes may reflect modulation of autophagy-associated regulatory networks at the gene expression level. However, these findings are based exclusively on mRNA measurements and should not be interpreted as direct evidence of functional autophagy activation or autophagic flux.

In contrast, molecule 4 treatment was associated with comparatively lower transcriptional changes in selected autophagy-related genes, including slight reductions in ATG6 (0.81-fold) and ATG7 (0.78-fold) expression relative to control conditions. This pattern may indicate comparatively limited transcriptional engagement of autophagy-associated regulatory pathways under these experimental conditions.

Similarly, AMBRA1, which participates in regulatory signaling processes associated with autophagy pathway control (Li et al. 2022; Sharif et al. 2016), showed increased transcriptional expression following molecule 6 treatment (2.12-fold) and comparatively reduced expression following molecule 4 treatment (0.69-fold). These findings further support differential transcriptional responses between the tested molecules rather than definitive functional pathway activation.

Overall, the transcriptional modulation observed for molecule 6 across apoptosis- and autophagy-associated genes may indicate broader engagement of cellular stress-response and cell death regulatory networks at the gene expression level. These findings support the potential of molecule 6 as an early-stage candidate molecule for further mechanistic and functional validation studies in pancreatic cancer models. Molecule 4 demonstrated comparatively lower transcriptional modulation across these gene sets under the same experimental conditions.

The present conclusions regarding autophagy are only derived from gene expression analysis. To confirm whether the observed transcriptional alterations correspond to functional autophagy activitiy, further research involving LC3-II accumulation, p62 degradation, and autophagic flux experiments will be necessary. Even though all mechanistic statements have been carefully revised to avoid any implication of confirmed protein-level activation, protein-based validation approaches, such as Western blotting and immunofluorescence analyses, are required to substantiate these findings and are planned for future investigations.

### In silico evaluations for target molecules

#### Docking studies

**Fig. 6:**
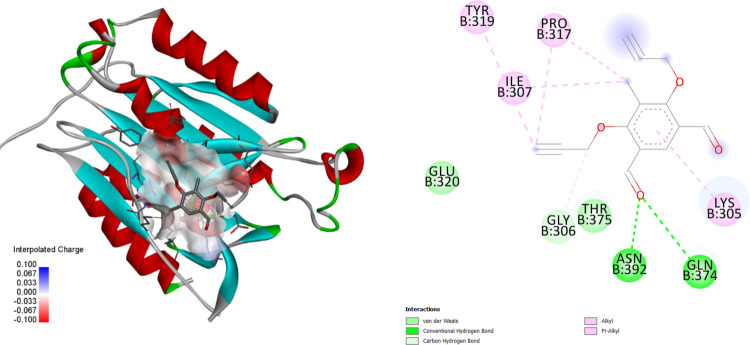
3D and 2D ligand–protein interactions of Caspase-8 (3H11) active site with molecule **6**

**Fig. 7:**
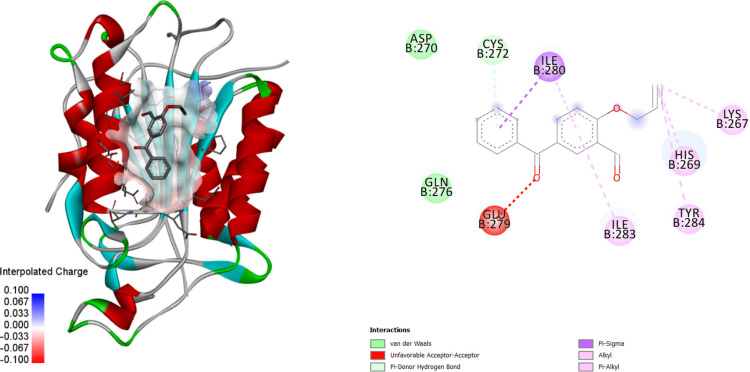
3D and 2D ligand–protein interactions of Caspase-8 (3H11) active site with molecule **4**

The molecular docking studies revealed valuable insights into the possible binding interactions of molecules **4** and **6** with apoptosis- and autophagy-related proteins such as caspase-8, ATG8, AMBRA1, and BAX, which were more expressed in studied genes. The comparative docking analysis of molecules **4** and **6** within the Caspase-8 (3H11) active site demonstrates notable differences in their binding orientations, interaction types, and overall stabilization patterns (Fig. 6 and 7). As shown in Fig. 6, molecule **6** fits deeply into the catalytic cleft, forming multiple stabilizing interactions including conventional hydrogen bonds with Gln-374, Thr-375, and Asn-392, as well as π–alkyl and alkyl contacts with Tyr-319, Pro-317, and Lys-305. These combined polar and hydrophobic interactions anchor the ligand efficiently, suggesting a strong affinity and optimal orientation within the enzyme’s active region. In contrast, molecule **4** exhibits a more superficial binding conformation, dominated by fewer hydrogen bonds (notably with Asp-319 and Tyr-370) and weaker hydrophobic contacts. The electrostatic surface visualization also reveals that molecule **4** occupies a less charged environment, implying a comparatively weaker electrostatic complementarity with the binding pocket. Collectively, these findings indicate that molecule **6** engages Caspase-8 through a more diverse and cohesive network of interactions, enhancing its potential to modulate apoptotic signaling, whereas molecule **4** shows a less favorable interaction profile consistent with lower binding stability and likely reduced biological activity.

**Fig. 8:**
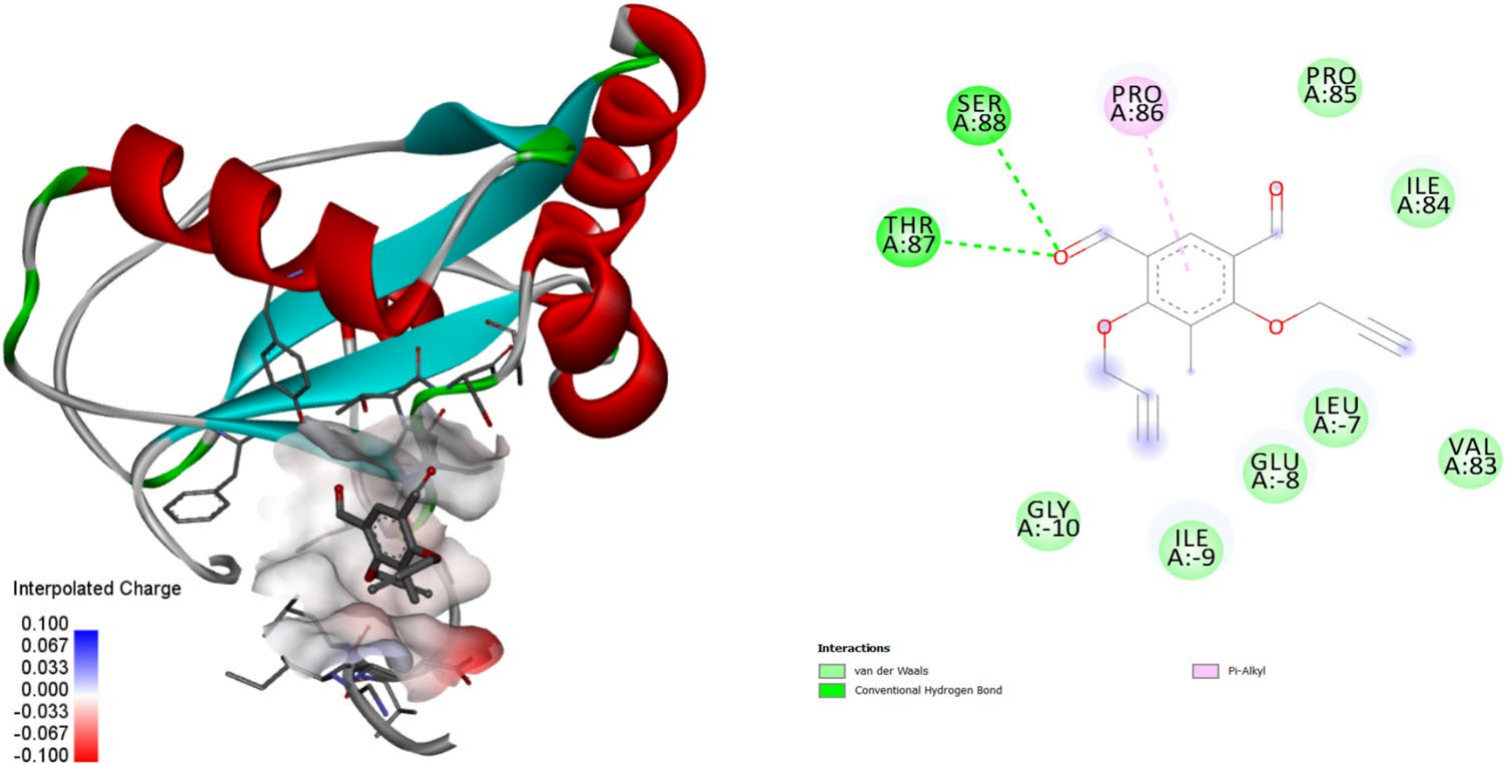
3D and 2D ligand–protein interactions of ATG-8 (6HB9) active site with molecule **6**

The molecular docking results of molecules** 4** and** 6** with the ATG8 (6HB9) protein reveal distinct differences in their binding affinities and interaction networks, reflecting their potential influence on autophagic signaling. As illustrated in Fig. 8, molecule **6** forms a stable complex within the ATG8 binding pocket through conventional hydrogen bonds with Thr-87 and Ser-88, complemented by π–alkyl interactions with hydrophobic residues such as Pro-85, Ile-84, and Leu-7. This combination of polar and nonpolar contact contributes to a compact and well-oriented binding mode, suggesting a strong affinity toward the active site. The electrostatic potential distribution indicates favorable charge complementarity, enhancing ligand stabilization within the moderately hydrophobic groove of ATG8.

**Fig. 9:**
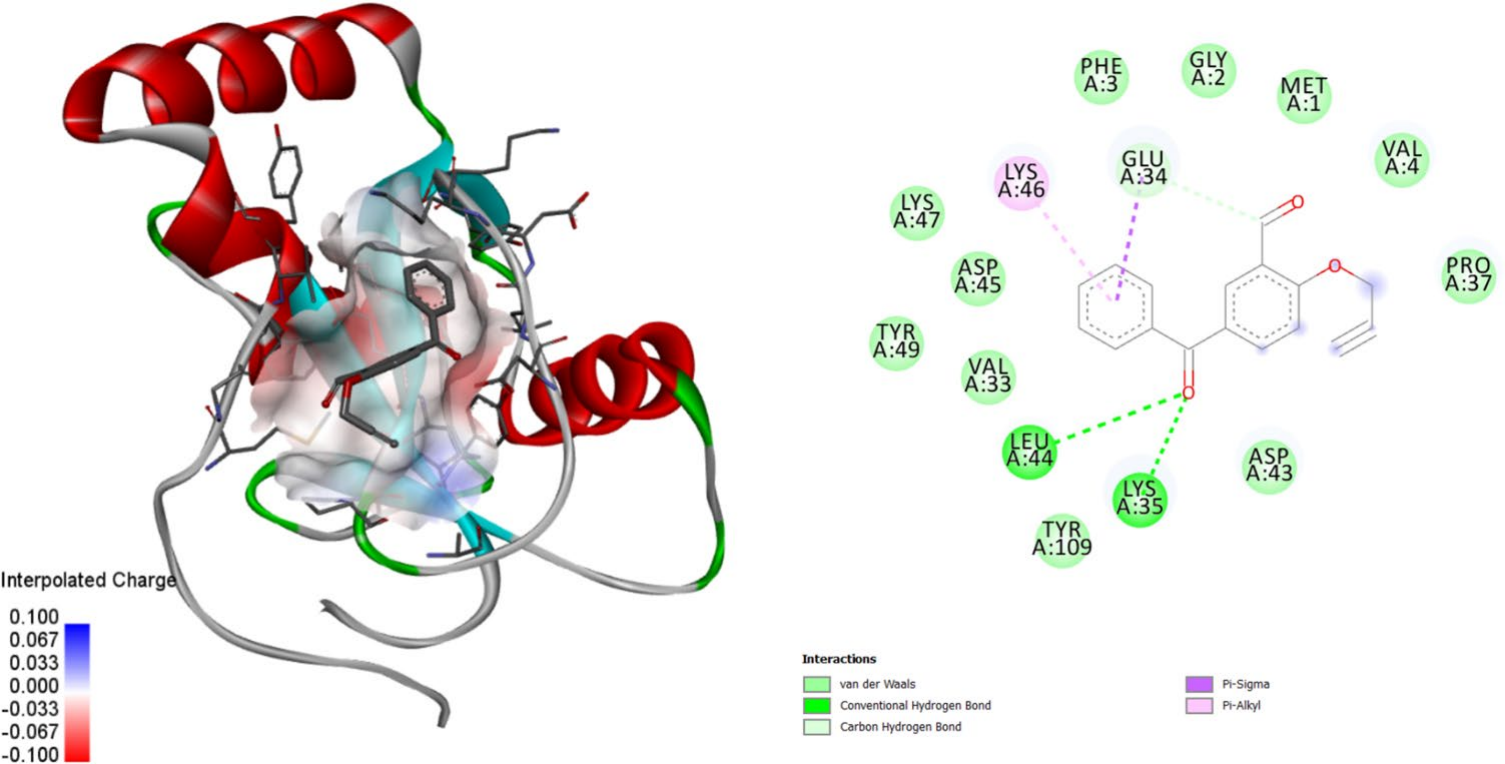
3D and 2D ligand–protein interactions of ATG-8 (6HB9) active site with molecule **4**

In contrast, molecule** 4** establishes fewer hydrogen bonds, particularly with Leu-46 and Lys-35, along with π–π and π–alkyl interactions (Fig. 9). Although these interactions are significant, the overall binding conformation of molecule** 4** appears more exposed, indicating a slightly reduced encapsulation within the binding pocket. This may correspond to lower overall stabilization energy compared to molecule **6**.

Collectively, these findings suggest that molecule** 6** interacts more efficiently with both hydrophilic and hydrophobic residues of ATG8, leading to enhanced complex stability and potentially stronger modulation of autophagy-related pathways. Molecule **4**, while still capable of hydrogen bonding, exhibits a less favorable interaction geometry, implying a comparatively weaker binding affinity and limited influence on ATG8-mediated processes.

**Fig. 10:**
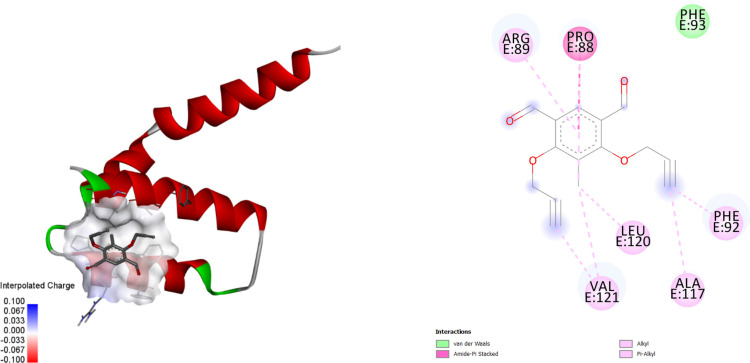
3D and 2D ligand–protein interactions of BAX (8G1T) active site with molecule **6**

**Fig. 11:**
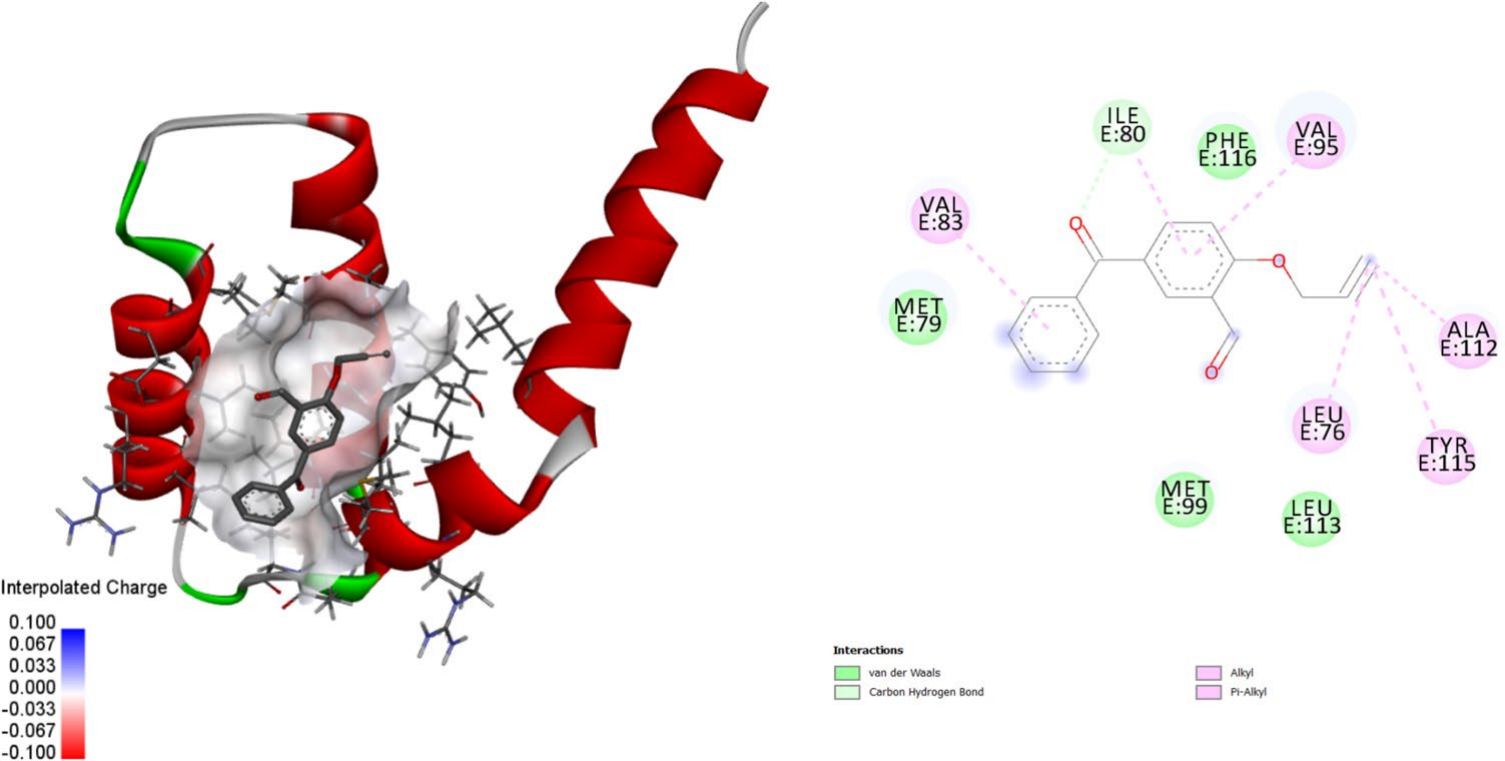
3D and 2D ligand–protein interactions of BAX (8G1T) active site with molecule **4**

The molecular docking analyses of molecules** 4** and **6** with the BAX (8G1T) protein provide critical insights into their potential roles in modulating apoptotic signaling through direct interactions with pro-apoptotic proteins. As illustrated in Figs. 10 and 11, both molecules occupy similar hydrophobic clefts of BAX, yet exhibit distinct interaction profiles that influence binding affinity and stability.

Molecule** 6** demonstrates a more deeply embedded orientation within the BAX binding cavity, stabilized primarily by amide–π stacked, π–alkyl and alkyl interactions with Pro88, Arg89, Phe92, and Leu120. These interactions suggest strong hydrophobic anchoring and aromatic stacking that enhance the conformational stability of the ligand–protein complex. The electrostatic surface representation reveals that molecule **6** is positioned within a moderately charged hydrophobic environment, enabling favorable Van der Waals and π-type contacts. Such interactions are known to reinforce protein conformational transitions associated with BAX activation, potentially promoting mitochondrial outer membrane permeabilization (MOMP) and apoptotic progression.

In contrast, molecule** 4** exhibits a broader but less deeply seated interaction network, involving Van der Waals and π–alkyl contacts with residues such as Met79, Val83, Leu113, and Phe116, alongside a carbon–hydrogen bond with Met99. Although these interactions indicate a stable binding pattern, their peripheral distribution suggests a relatively lower degree of pocket penetration compared to molecule **6**. This may translate to a weaker stabilization effect on the BAX activation interface.

Overall, the comparative analysis highlights that molecule** 6** forms a more compact and energetically favorable complex with BAX, dominated by strong aromatic and amide–π interactions, whereas molecule** 4** engages the protein primarily through less specific hydrophobic contacts. These findings imply that molecule **6** may possess a greater potential to modulate BAX-mediated apoptotic signaling, aligning with its stronger docking score and probable pro-apoptotic activity observed in related assays.

**Fig. 12:**
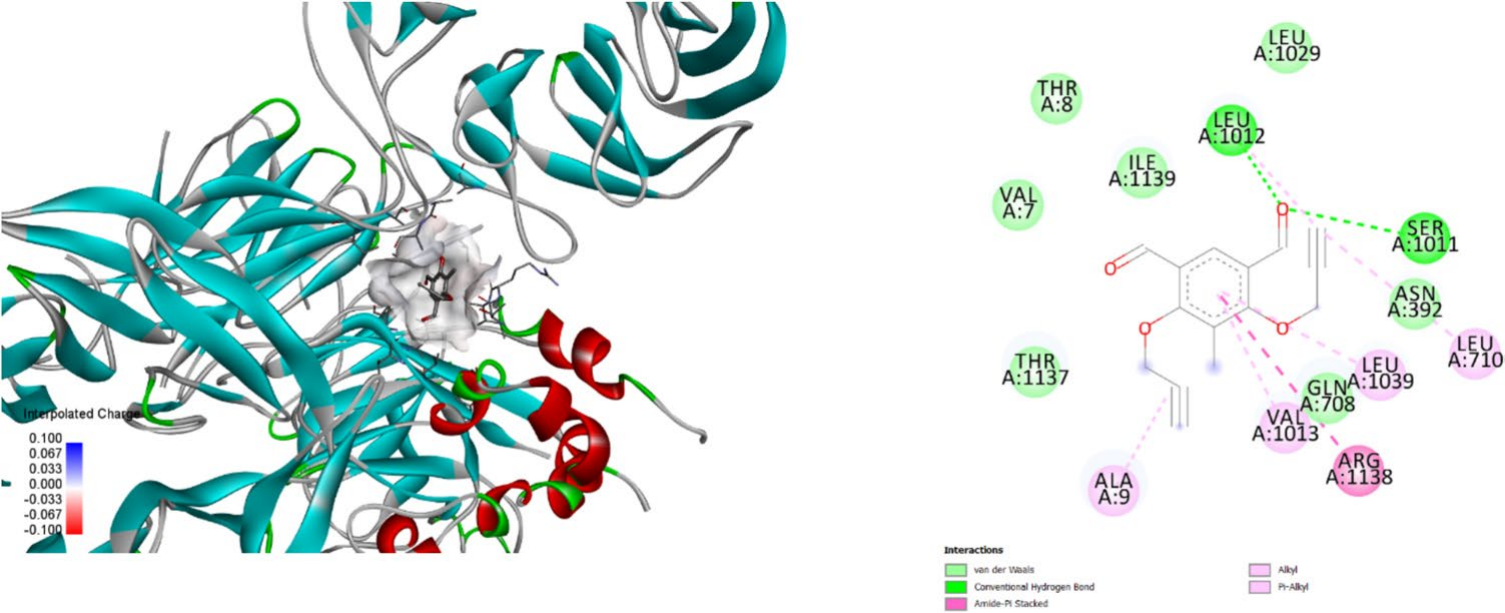
3D and 2D ligand–protein interactions of AMBRA1 (8WQR) active site with molecule **6**

The molecular docking analysis of molecules **4** and** 6** with AMBRA1 (8WQR) highlights significant differences in their binding affinities and interaction profiles, which may influence their roles in regulating autophagy. As shown in Fig. 12, molecule **6** fits deeply into the AMBRA1 binding cavity and forms multiple stabilizing interactions, including hydrogen bonds with Ser1011 and Leu1012, and hydrophobic or π–alkyl contacts with Val1013, Leu 1039, Arg1138, Ala 9, and Leu 710. These interactions create a balanced network of polar and nonpolar forces, resulting in a compact and energetically favorable binding conformation. The electrostatic potential distribution also indicates strong complementarity between the ligand and the protein surface, supporting its higher stability within the active site.

**Fig. 13:**
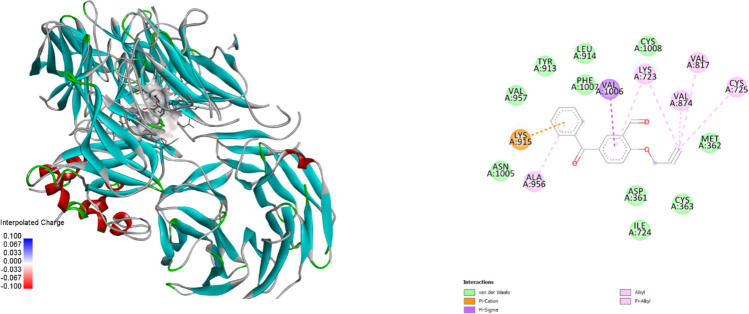
3D and 2D ligand–protein interactions of AMBRA1 (8WQR) active site with molecule **4**

In contrast, molecule** 4** displays a more extended and surface-oriented binding mode, dominated by π–cation and π–σ interactions with Lys915 and Phe1006, as well as hydrophobic contacts with Met362, Val874, Cys725, Lys 723, and Ala 956 (Fig. 13). While these interactions contribute to moderate binding affinity, the limited number of hydrogen bonds suggests weaker polar stabilization and a less optimal fit within the binding groove.

Overall, the data suggest that molecule** 6** exhibits a stronger and more specific interaction pattern with AMBRA1, integrating hydrogen bonding, hydrophobic, and aromatic interactions that enhance its affinity and potential biological relevance. In comparison, molecule** 4** interacts primarily through nonpolar forces, indicating a lower degree of structural stabilization and potentially reduced capacity to modulate AMBRA1-dependent autophagic mechanisms.

The docking energy values summarized in Table 3 demonstrate that molecule **4** exhibits stronger binding affinities toward all four target proteins compared to molecule **6**. Among the targets, the most favorable binding was observed between molecule** 4** and ATG8 (6HB9, − 6.38 kcal/mol), indicating a particularly high affinity for this autophagy-related protein. This is followed by AMBRA1 (8WQR, − 5.12 kcal/mol) and BAX (8G1T, − 4.92 kcal/mol), suggesting that molecule **4** can interact efficiently with both autophagic and apoptotic regulators. In contrast, molecule** 6** shows comparatively weaker affinities, with binding energies ranging from − 3.53 to − 4.29 kcal/mol, implying less stable complex formation and reduced interaction strength. Although molecule 4 exhibits higher advantageous docking energies, molecule 6 establishes a more intricate and clinically pertinent contact network, especially with apoptosis-regulating proteins. Therefore, binding mode dynamics rather than binding affinity alone are probably responsible for molecule 6’s biological action.

**Table 3:** Binding energy values for molecules **4** and **6** using selected proteins

Binding energy	Molecule 4	Molecule 6
8WQR	−5.12	−4.18
6HB9	−6.38	−4.29
3H11	−4.25	−3.71
8G1T	−4.92	−3.53

The comparative analysis of molecules **4** and **6,** based on both radar plots and selected physicochemical descriptors, provides insight into their drug-likeness and potential behavior in biological systems (SwissADME 2017).

**Fig. 14:**
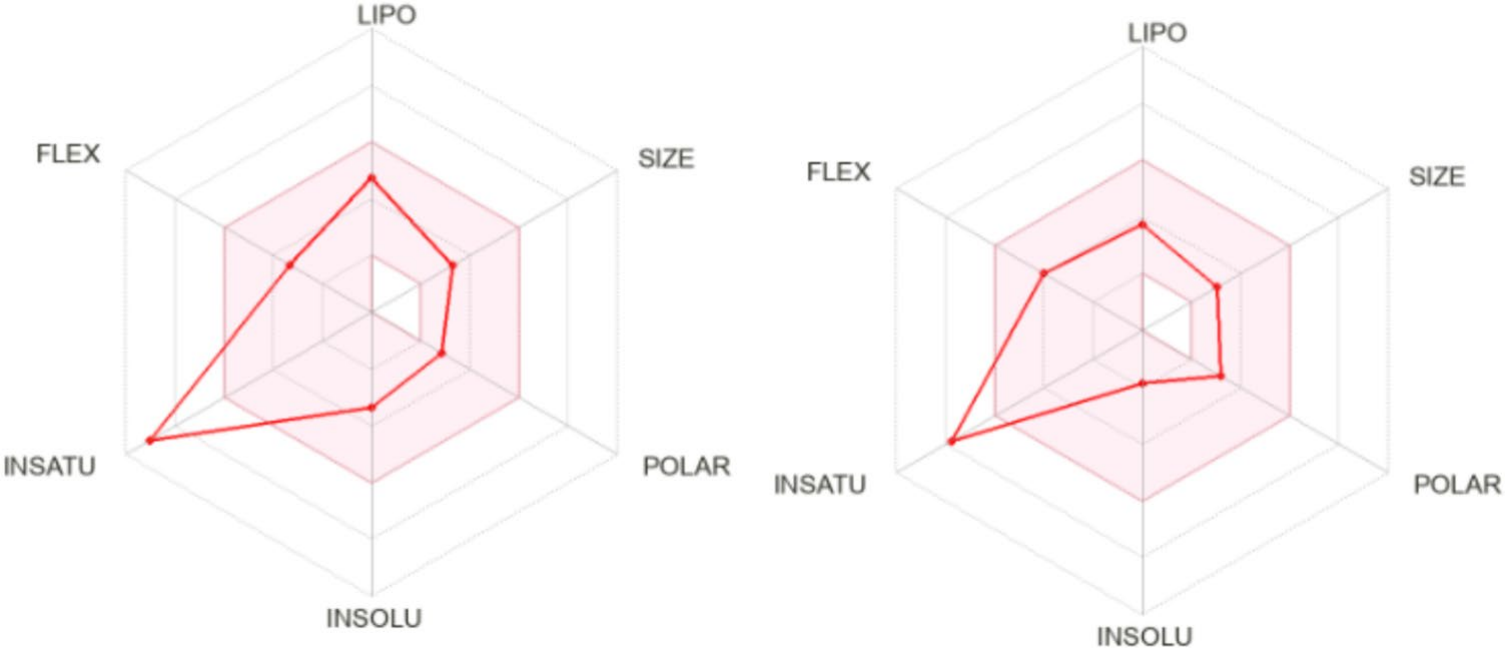
Radar graphes for molecules **4** and **6**. Left for molecule** 4** and right for molecule **6**

The radar graphs (Fig. 14) show that both molecules have similar profiles in terms of lipophilicity (LIPO) and molecular flexibility. However, molecule **4** has a significantly greater level of unsaturation (INSATU), which might indicate a more stiff and planar molecular framework. This might affect target binding by increasing π-π interactions or reducing conformational entropy loss during binding. Furthermore, molecule **4** seems somewhat bigger in size (SIZE) and less polar (POLAR), perhaps increasing membrane permeability but at the expense of diminished water solubility (INSOLU), as seen by its radar profile.

In terms of numerical properties (Table 4), molecule **4** has a higher estimated LogP (2.95 vs. 1.85), indicating a more lipophilic nature that may facilitate passive diffusion across lipid membranes. This might be beneficial for intracellular targets, but it may need close monitoring of metabolic stability and off-target lipid interactions. The molecular refractivity (MR), which corresponds with molecular size and polarizability, is similarly larger for molecule **4** (75.97 vs. 65.75), supporting the radar plot finding of increased size and stiffness.

**Table 4:** Some physicochemical parameters for molecules **4** and **6**

	Molecule 4	Molecule 6
CLog P	2.95	1.85
MR	75.97	65.75
TPSA	43.47 A	52.60
Lipinski Rule of Five	0 violation	0 violation

Molecule **6** has a slightly bigger Topological Polar Surface Area (TPSA) (52.60 Å^2^ vs. 43.47 Å^2^), although both molecules are still within the desirable range for oral bioavailability (< 90 Å^2^). This increase in polarity may result in increased solubility and lower central nervous system (CNS) penetration, which may be desired depending on the therapeutic situation. Importantly, both molecules perfectly adhere to Lipinski’s Rule of Five, with no breaches detected, indicating their potential as orally accessible therapeutic candidates.

To summarize in silico research, molecule **4**, with its higher lipophilicity and lower polarity, may have superior membrane permeability but maybe poorer solubility. Molecule **6**, on the other hand, has a more balanced hydrophobicity-polarity profile, which might lead to improved solubility and lower nonspecific binding. The eventual decision amongst these candidates would be based on the targeted biological target, needed pharmacokinetic profile, and therapeutic indication.

#### MD simulation for molecule 4 and 6 with selected proteins

The molecular dynamics simulations across all tested complexes demonstrate that both molecule **4** and molecule **6** establish highly stable and structurally robust interactions with the 8WQR, 8G1T, 6HB9, and 3H11 protein targets (Figure S43-S50). A consistent hallmark of these simulations is the rapid attainment of a Root Mean Square Deviation (RMSD) plateau, ranging from a remarkably rigid 1.0–1.6 Å in the 6HB9 complexes to a stable 3.0–6.0 Å in others, signifying successful equilibration and the preservation of the global protein fold throughout the trajectories. While molecule **6** often undergoes a good initial reorientation to achieve an optimized binding pose, molecule **4** tends to maintain lower overall deviation, yet both ligands exhibit restricted internal mobility once stabilized, as evidenced by ligand Root Mean Square Fluctuation (RMSF) values consistently below 1.5 Å. The structural integrity is further reinforced by the persistent or dynamically maintained hydrogen-bonding networks and favorable nonpolar interactions, which collectively anchor the ligands within the binding pockets. The localized rigidity observed at the binding interfaces, contrasted with the natural flexibility of peripheral loops, confirms that these molecules act as high-affinity binders that thermodynamically stabilize their respective targets, making them promising lead candidates for further therapeutic development. We want to summarize the MD simulation results with key comparative highlights:

1. **Structural Rigidity:** Molecule 4 generally exhibits lower RMSD values (1.0–2.4 Å) compared to Molecule 6 (3.0–6.0 Å), indicating a more immediate structural fit with the target backbones.
2. **Interaction Drivers:** While Molecule 4 relies heavily on high-occupancy, persistent hydrogen bonds, Molecule 6 stability is often governed by a sophisticated combination of nonpolar dispersive forces and transient but dynamic hydrogen bonding.
3. **Target Specificity:** Both molecules show exceptional stability with 6HB9 and 8G1T, characterized by the lowest RMSD and RMSF values in the set, identifying these as particularly favorable target-ligand pairings.

### Hen’s egg test on chorioallantoic membrane (HET-CAM)

**Fig. 15:**
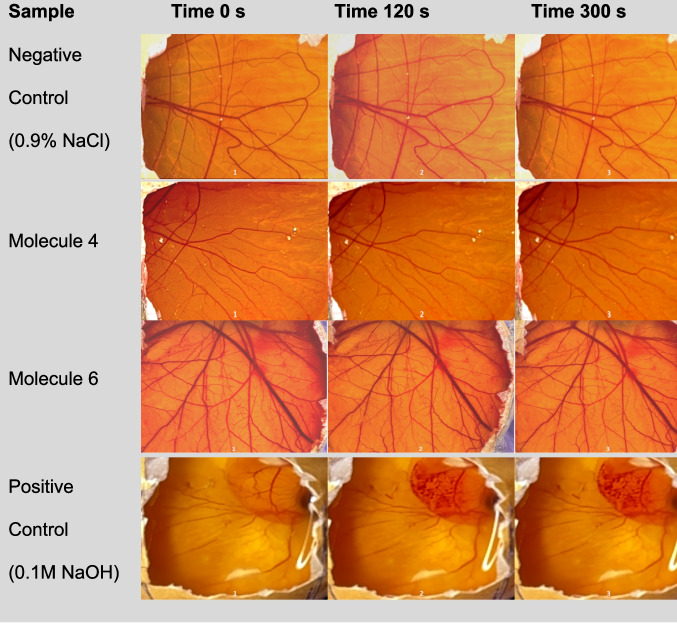
HET-CAM test results showing the appearance of the chorioallantoic membrane (CAM) at defined time intervals following exposure to Molecule 4 and Molecule 6 (*n* = 3)

In this study, the HET-CAM assay was used to assess the irritation potential of molecule **4** and molecule **6** by monitoring the onset of vascular damage, including hemorrhage, vascular lysis, and coagulation. The reactions were scored according to the ICCVAM-recommended irritation scoring system, as presented in Table 5 and Fig. 15. Based on the calculated IS values, both molecule **4** and molecule **6** were classified as non-irritant at the membrane surface (Table 6).

**Table 5:** HET-CAM irritation parameters and scores of Molecule 4 and Molecule 6

Sample	tH (s)	tL (s)	tC (s)	IS Score	Irritation Category (ICCVAM)
Negative Control (0.9% NaCl)	301	301	301	0.00	Non-irritant
Molecule 4	301	301	301	0.00	Non-irritant
Molecule 6	301	301	301	0.00	Non-irritant
Positive Control (0.1 M NaOH)	8.75	13.75	14.63	18.9	Severe irritant

**Table 6:** Primer sequences used for quantitative RT-PCR analysis

Gen	Primer Sequences (5′→3’)	Product Size (bp)	Accession Number
Bax	F:5’-GATGGACGGGTCCGGGG R: 5’- CGATCCTGGATGAAACCCTGA	102	NM_004324.4
Bad	F:5’- CCCAGAGTTTGAGCCGAGTG R:5’- CCCATCCCTTCGTCGTCCT	249	NM_004322.3
Casp3	F: 5’- ATGGAAGCGAATCAATGGAC R: 5’- AGTTTCTGAATGTTTCCCTGAG	177	NM_004346.4
Casp8	F: 5’- GATGTTATTCCAGAGACTCCAG R: 5′- GGTAGGTAATCAGCAAATCCA	110	NM_001080125.2
Casp9	F: 5’- CTTCGTTTCTGCGAACTAACAGG R: 5’- GCACCACTGGGGTAAGGTTT	75	NM_001229.5
Bcl2	F: 5’- GGATAACGGAGGCTGGGATG R: 5’- TGACTTCACTTGTGGCCCAG	156	NM_000633.3
Bcl-xL	F: 5’- AGCTTTGAACAGGTAGTGAATGA R: 5′- TTCCCATAGAGTTCCACAAAAGT	227	NM_138578.3
ATG6	F: 5’- GGTTTTTCTGGGACAACAAGTTT R: 5′- AACTGGGTTTTGATGGAATAGGA	184	NM_003766.5
ATG7	F: 5’-TTTTAGTAGTGCCTTGGATGTTG R: 5′-AGCAGAGTCACCATTGTAGTAAT	121	NM_006395.3
ATG12	F: 5’- TTACGGATGTCTCCCCAGAAAC R: 5′- TTGGATGGTTCGTGTTCGCT	182	NM_004707.4
AMBRA1	F: 5’-GGACCCCTTCCTTTATTCCTGT R: 5′-GCATTCTTTTCTGGGACAACCT	239	NM_001267782.2
GAPDH	5’- AGGCCTGCTTTTAAC 5′- CCCCACTTGATTTTG	206	NM_002046.7

The HET-CAM results showed that neither Molecule 4 nor Molecule 6 induced hemorrhage, vascular lysis, or coagulation during the 300-s observation period, resulting in an irritation score of zero for both test samples. The absence of any acute vascular or membrane response indicates that the tested concentrations are well tolerated at the CAM surface. The lack of irritation also suggests that the formulation components, including the vehicle, do not contribute to adverse membrane reactions. This non-irritant profile supports the suitability of these molecules for further biological and pharmacological studies, as no immediate irritation-related limitations are expected at this stage.

In the context of our study, HET-CAM was used to complement our in vitro findings by evaluating the compound’s immediate biocompatibility and irritation potential on a highly vascularized membrane, thereby addressing a key translational objective, early safety screening prior to more resource-intensive animal studies. This assay therefore strengthens the overall study by linking mechanistic efficacy data with an initial, biologically relevant tolerability assessment.

## Conclusion

The present study provided compelling evidence for the therapeutic promise of molecule 6 as a hit candidate in the treatment of pancreatic cancer, a malignancy marked by limited treatment options and poor prognosis. Among the nine propargylated derivatives evaluated, molecule 6 emerged as one of the most potent molecules, exhibiting significant cytotoxic activity against the PANC-1 pancreatic cancer cell line. Mechanistically, molecule 6 demonstrated a remarkable ability to orchestrate a dual-modality effect by concurrently modulating transcriptional responses associated with apoptosis and autophagy, two critical and interrelated pathways in cancer regulation. The pronounced upregulation of apoptotic markers, Bax, Bad, Caspase-8, and Caspase-9, along with substantial transcriptional upregulation of autophagy-related genes, ATG6, ATG7, ATG8, and ATG12, suggests a coordinated cytotoxic mechanism that disrupts tumor cell survival through multiple cellular stress responses. Importantly, these observations are derived from gene expression analyses and should be interpreted as transcriptional engagement of apoptosis- and autophagy-related regulatory networks rather than direct evidence of functional pathway activation. This dual-action profile not only amplifies the cytotoxic impact but also provides a strategic advantage in overcoming resistance mechanisms frequently observed in pancreatic cancer therapy.

Furthermore, the concurrent transcriptional upregulation of both pro-apoptotic (e.g., Bax, Bad) and anti-apoptotic (e.g., Bcl-XL) regulators observed in this study is consistent with adaptive cellular stress-response signaling, where both survival- and death-associated pathways may be transiently activated prior to definitive cell fate determination.

In contrast, molecule 4, while displaying moderate cytotoxicity, demonstrated a more limited interaction with key molecular targets, indicating potential room for structural optimization to enhance its therapeutic value. Although molecules 4 and 6 exhibited cytotoxic effects against PANC-1 cells (IC_50_ = 65.7 µM and 80.6 µM, respectively), these values are within the high-micromolar range, indicating only moderate potency. PANC-1 cells are known to be relatively drug-resistant, and MTT assays assess metabolic activity rather than direct cell death. Therefore, additional validation using orthogonal viability assays (e.g., CellTiter-Glo), colony formation assays, and direct measurements of apoptosis and autophagy flux will be planned. Furthermore, in ovo HET-CAM assay confirmed that both Molecule 4 and Molecule 6 exhibit a non-irritant profile, as neither molecule triggered hemorrhage, vascular lysis, nor coagulation, resulting in an irritation score of zero. This absence of acute membrane or vascular toxicity supports the biological tolerability of the molecules and strengthens their suitability for further pharmacological and in vivo investigation.

These findings contribute valuable insights into the structure–activity relationships of propargylated scaffolds and highlight the significance of targeting multiple cell death pathways for improved therapeutic efficacy. Moving forward, our research will prioritize the structural refinement of molecule 6, comprehensive pharmacokinetic profiling, and in vivo validation in relevant pancreatic cancer models. The ultimate goal is to transition molecule 6 into a viable preclinical candidate, with the potential to offer a novel and effective approach to pancreatic cancer treatment and moving it to the next step by the in-vivo studies.

## Experimental section

### Chemistry

#### General procedure for preparation of propargylated salicylaldehyde and one propargylated indole aldehyde derivatives (1–9)

**Scheme 2.**
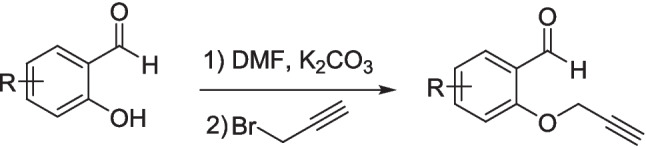
General procedure for preparation of propargylated molecules

To a round bottom flask was added, (10 mmol) of salicylaldehyde or indole aldehyde dissolved in 5 mL of dimethyl formaldehyde (DMF). To that, (20 mmol) potassium carbonate (K_2_CO_3_) was added then mixed at room temperature for at least an hour (Scheme 2). Next, (16 mmol) propargyl bromide was added drop by drop mixed with the solvent, while the mixture is still mixing at previous conditions and continued at room temperature overnight. Extraction involved brine and ethyl acetate, then dried with magnesium sulphate (MgSO_4_) as a drying agent, after which TLC confirmation was done. The solvent is removed under reduced pressure conditions. The residue was purified by column chromatography (mobile phase: hexane; ethyl acetate 20:1, stationary phase: silica gel).

##### 2-(prop-2-yn-1-yloxy)benzaldehyde (1)

**Yield: 90%** ^1^H NMR (400 MHz, CDCl_3_) *δ* 10.41 (s, 1H), 7.79 (dt, *J* = 1.4 Hz, 7.7 Hz, 1H, Ar–H), 7.50 (m, 1H, Ar–H), 7.03 (m, 2H, Ar–H), 4.76 (d*, J* = 2.4 Hz, 2H, CH_2_), 2.5(t, *J* = 2.4 Hz, 1H, CH), ^13^C NMR (101 MHz, CDCl_3_) *δ* 188.6, 158.8, 134.7, 133.89, 127.6, 124.5, 120.7, 112.2, 76.6, 75.5, 55.4.[38].

##### 5-nitro-2-(prop-2-yn-1-yloxy)benzaldehyde (2)

**Yield: 75%** ^1^H NMR (400 MHz, CDCl_3_) *δ* 10.35 (s, 1H), 8.56 (m, 1H, Ar–H), 8.35 (m, 1H, Ar–H), 7.25 (s, 1H, Ar–H), 4.93 (t, *J* = 2.4 Hz, 2H, CH_2_), 2.64 (t, *J* = 2.4 Hz, 1H, CH).^13^C NMR (101 MHz, CDCl_3_) *δ*187.1, 163.7, 142.1, 130.5, 125.1, 124.4, 113.9, 77.9, 76.3, 57.2 (Vedachalam et al. 2011).

##### 3-methoxy-2-(prop-2-yn-1-yloxy)benzaldehyde (3)

**Yield: 88%** ^1^H NMR (400 MHz, CDCl_3_) *δ* 10.31 (s, 1H), 7.31 (dt, *J* = 5.2,4.3 Hz, 1H, Ar-1H), 7.05 (m, 2H, Ar–H), 4.76(dd*, J* = 0.8, 2.5 Hz, 2H), 3.77(d, *J* = 1.2 Hz, 3H), 2.42(t, *J* = 2.4 Hz, 1H),^13^C NMR (101 MHz, CDCl_3_) *δ* 190.4, 152.8, 149.4, 131.0, 124.9, 118.7, 117.8, 78.3, 77.0, 60.8, 56.0, 55.9. (Wang et al. 2019).

##### 5-benzoyl-2-(prop-2-yn-1-yloxy)benzaldehyde (4)

**Yield 60%** ^1^H NMR (400 MHz, CDCl_3_) *δ* 10.41 (s, 1H), 8.20 (d, *J* = 2.3 Hz, 1H, Ar–H), 8.07 (m, 1H, Ar–H), 7.65 (m, 2H, Ar–H), 7.50 (m, 1H, Ar–H), 7.39 (m, 2H, Ar–H), 7.18 (d, *J* = 8.7 Hz, 1H, Ar–H), 4.86 (d, *J* = 2.4 Hz, 2H, CH_2_), 2.56 (t, *J* = 2.4 Hz, 1H, CH).^13^C NMR (101 MHz, CDCl_3_) *δ* 193.7, 187.6, 161.4, 136.4, 136.3, 136.2, 131.5, 130.2, 130.0, 128.7, 127.5, 123.5, 112.2, 76.2, 75.9, 75.5, 55.6. HRMS (ES) [M + H]; *m*/*z:* Calculated for C_17_H_13_O_3_: 265.0865 Found *265.0846.*

##### 3-methyl-2,4-bis(prop-2-yn-1-yloxy)benzaldehyde (5)

**Yield 60%** ^1^H NMR (400 MHz, CDCl_3_) *δ* 10.23 (d, *J* = 0.7 Hz, 1H), 7.70 (dd, *J* = 8.7, 0.7 Hz, 1H, Ar–H), 6.84 (d, *J* = 8.7 Hz, 1H, Ar–H), 4.73 (d, *J* = 2.4 Hz, 2H, CH_2_), 4.60 (d, *J* = 2.4 Hz, 2H, CH_2_), 2.49 (t, *J* = 2.4 Hz, 1H, CH), 2.46 (t, *J* = 2.4 Hz, 1H, CH), 2.15 (s, 3H, CH_3_).^13^C NMR (101 MHz, CDCl_3_) *δ* 188.5, 160.7, 158.9, 126.7, 123.4, 120.1, 107.3, 76.9, 76.7, 76.2, 75.2, 61.4, 55.3, 8.2. HRMS (ES) [M + H]; *m*/*z:* Calculated for C_14_H_12_O_3_: 229.0865 Found 229.08478.

##### 5-methyl-4,6-bis(prop-2-yn-1-yloxy)isophthalaldehyde (6)

**Yield 70%** ^1^H NMR (400 MHz, CDCl_3_)* δ* 10.25 (d, *J* = 1.1 Hz, 2H), 8.21 (s, 1H, Ar–H), 4.72 (d, *J* = 2.4 Hz,, 4H, CH_2_), 2.49 (t,, *J* = 2.4 Hz,, 2H, CH), 2.29 (s, 3H, CH_3_).^13^C NMR (101 MHz, CDCl_3_) 187.8, 162.7, 128.5, 127.1, 126.4, 76.8, 76.3, 61.4, 9.1. HRMS (ESI) [M + H]; Calculated for C_15_H_13_O_4_: 257.0814;Found: 257.0810.

##### 2-(prop-2-yn-1-yloxy)−1-naphthaldehyde (7)

**Yield 65%**^1^H NMR (400 MHz, CDCl_3_) *δ* 10.90 (s, 1H), 9.27 (dd, *J* = 1.2, 8.7 Hz, 1H, Ar–H), 8.06 (d, *J* = 9.1 Hz, 1H, Ar–H), 7.78 (d, *J* = 7.4 Hz, 1H, Ar–H), 7.63 (m, 1H, Ar–H), 7.44 (m, Ar–H), 7.37 (d, *J* = 9.1 Hz, 1H, Ar–H), 4.94 (d, *J* = 2.4 Hz, 2H, CH_2_), 2.57 (t, 2.4 Hz, 1H, CH).^13^C NMR (101 MHz, CDCl_3_) *δ* 192.0, 161.9, 137.3, 131.5, 129.9, 129.1, 128.3, 125.2, 125.1, 118.1, 114.0, 77.7, 76.8, 57.4. (Wang et al. 2019).

##### 4-(prop-2-yn-1-yloxy)isophthalaldehyde (8)

**Yield 80%** ^1^H NMR (400 MHz, CDCl_3_) δ 10.42 (d, *J* = 1.2 Hz, 1H), 9.89 (s, 1H), 8.29 (dd, *J* = 2.3, 1.2 Hz, 1H, Ar–H), 8.07 (ddd, *J* = 8.6, 2.2, 0.9 Hz, 1H, Ar–H), 7.2 (ddd, *J* = 8.6, 2.2, 0.9 Hz, 1H, Ar–H), 4.88 (d, *J* = 2.5 Hz, 2H, CH_2_), 2.58 (t, *J* = 2.4 Hz, 1H, CH). ^13^C NMR (101 MHz, CDCl_3_) δ 190.1, 188.3, 163.6, 135.4, 131.9, 130.4, 125.4, 113.7, 77.5, 76.7, 56.7. (Chen et al. 2019).

##### 1-(prop-2-yn-1-yl)−1H-indole-2-carbaldehyde (9)

**Yield 90%** ^1^H NMR (400 MHz, CDCl_3_) δ, 9.79 (s, 1H), 7.66 (dt, *J* = 8.1, 1.0 Hz, 1H, Ar–H), 7.45 (dq, *J* = 8.5, 1.0 Hz, 1H, Ar–H), 7.38 (ddd, *J* = 8.4, 6.9, 1.1 Hz, 1H, Ar–H), 7.20 (d, *J* = 0.9 Hz, 1H, Ar–H), 7.14 (ddd, *J* = 8.0, 6.8, 1.0 Hz, 1H, Ar–H), 5.36 (d, *J* = 2.6 Hz, 2H, CH_2_), 2.18 (t, *J* = 2.5 Hz, 1H, CH).^13^C NMR (101 MHz, CDCl_3_) δ 182.7, 140.1, 134.5, 127.4, 126.7, 123.6, 121.6, 118.7, 110.8, 78.2, 72.5, 33.9. (Guven et al. 2015).

### Molecular biology

#### Cell culture

PANC-1 cells were maintained in Dulbecco’s Modified Eagle Medium (DMEM) (ThermoFisher Scientific, USA; 11,995,065,) containing 10% FBS (Gibco, USA; A5256801), 100 units/mL penicillin, and 0.1 mg/mL streptomycin (Gibco, USA; 151–40–122) in 5% CO_2_ humidified air at 37 °C. (Luo et al. 2024).

#### MTT cytotoxicity assay

Cytotoxic effects were evaluated using the MTT [3-(4,5-dimethylthiazol-2-yl)−2,5-diphenyltetrazolium bromide] assay, and the half-maximal inhibitory concentrations (IC₅₀) were calculated for each molecule in PANC-1 (human pancreatic ductal adenocarcinoma) cells (Taspinar et al. 2013).

All synthesized molecules were prepared under sterile conditions within a Class II biosafety cabinet designated for cell-culture work. Each molecule was dissolved in 1 mL of dimethyl sulfoxide (DMSO; Sigma, D2650) to generate primary stock solutions, with stock concentrations adjusted according to the molecular weights of the individual molecules. All subsequent dilutions were prepared freshly in complete DMEM medium immediately before use to ensure chemical stability during treatment. Preliminary cytotoxicity assessments were performed using high-concentration working solutions (between 200–500 µM) to determine the upper cytotoxic thresholds of the molecules. Based on these initial evaluations, refined dose–response experiments were conducted using a concentration series of 10, 25, 50, 75, 100, 150, 200, 300, 400, and 500 µM. For all treatment conditions, the final concentration of DMSO did not exceed 0.1% (v/v), minimizing solvent-related cytotoxicity. All reagents and solvents were of molecular-biology grade and verified as suitable for cell-culture applications. Cells were trypsinized and seeded at a density of 1 × 10^4^ cells per well in 96-well plates. After a 24-h incubation at 37 °C in a humidified atmosphere with 5% CO₂, cells were serum-starved for 8 h to synchronize the cell cycle prior to treatment. PANC-1 cells were treated with different concentrations of prepared molecules, and incubations continued for 72 h. Following incubation, the medium was discarded, and MTT solution (0.5 mg/mL in fresh medium) was added to each well. Plates were incubated for 3 h at 37 °C to allow for formazan crystal formation. The medium was then removed, and 100 μL of lysis buffer [89% Isopropanol (Merck, 109,634); 11% Triton X-100 (Sigma,X100) and 1% HCl (Merck, H9892, 0.1 N)] was added to dissolve the formazan crystals. Absorbance was measured at 570 nm using a microplate spectrophotometer. In addition, after identifying the active molecules in PANC-1 cells, Molecule 4 and Molecule 6 were further tested for their cytotoxic effects in PC-3 (prostate adenocarcinoma), A549 (lung adenocarcinoma), and hDF (healthy human dermal fibroblast) cell lines. These assays were performed using the same MTT protocol above to evaluate cancer-selective activity and potential effects on non-malignant cells. All cytotoxicity experiments were performed in three independent trials, each containing four technical replicates per concentration. Dose–response curves and IC_50_ values were determined using nonlinear regression analysis in GraphPad Prism. To verify that DMSO did not influence IC₅₀ measurements, we performed a DMSO-only cytotoxicity control in PANC-1 cells (24 h, 48 h, 72 h). Viability remained 98–110% at 0.1% DMSO, confirming that this concentration is non-toxic.

The final DMSO concentration in all treatments was consistently maintained at ≤ 0.1%. We also included a vehicle control containing 0.1% DMSO, and no reduction in cell viability was observed compared with the untreated control, confirming that DMSO at this concentration did not contribute to the observed IC_50_ values. No turbidity, precipitation, or visible particle formation was detected at any tested dose (For details of screening of DMSO dose, see Figure S42).

#### Total RNA isolation and cdna synthesis

Based on the MTT assay results, two molecules with the lowest IC₅₀ values (Molecule 4 and Molecule 6) were selected for further molecular analysis. To investigate the effects of these molecules on apoptotic and autophagic gene expression, total RNA isolation and cDNA synthesis were performed. PANC-1 cells were seeded into 25 cm^2^ flasks (Greiner bio-one) (5 × 10^5^ cells per flask) and allowed to adhere for 24 h at 37 °C in a humidified 5% CO₂ atmosphere. Cells were then treated with the selected molecules at their IC₅₀ concentrations to ensure a biologically relevant exposure reflective of their cytotoxic potency. Following 72 h of treatment, total RNA was isolated using a TRIzol-based extraction method involving chloroform–isoamyl alcohol. RNA integrity was evaluated by 1% agarose gel electrophoresis, while RNA concentration and purity were assessed spectrophotometrically to confirm that the RNA was suitable for subsequent analyses. High-quality RNA samples were subsequently used for cDNA synthesis employing the High-Capacity cDNA Reverse Transcription Kit (Applied Biosystems™, Cat. No. 4368814) according to the manufacturer’s instructions.

#### Real-time polymerase chain reaction (RT-PCR)

Gene expression levels of apoptotic and autophagy-related markers were quantified by real-time PCR using the FastStart SYBR Green Master Mix (Roche, 064027112001). Primer sequences and amplicon sizes for all target genes are listed in Table 4. Each biological sample was analyzed in triplicate to ensure technical reproducibility, and Glyceraldehyde 3-phosphate dehydrogenase (GAPDH) was used as the internal reference gene for normalization (Radonić et al. 2004; Barber et al. 2005; Xu et al. 2020; Ungefroren et al. 2022). Following amplification, melting-curve analysis was performed to verify product specificity (See supporting information, Figure S19-S38). In the present study, the stability of GAPDH expression under treatment conditions was verified by comparing Ct values between control and compound-treated groups, and no significant variation was observed. In addition, consistent amplification and single-peak melt curves further supported the stability and suitability of GAPDH as a reference gene across all experimental conditions (see Supporting Information).

Relative gene expression differences were calculated using the 2⁻ΔΔCt method, following the approach of (Livak and Schmittgen 2001) and results were expressed as mean ± SD.

#### Statistical analysis

Comparisons of continuous variables across three independent groups, including a control group, were performed using one-way ANOVA. When a significant difference was detected, Tukey’s Honestly Significant Difference (HSD) test was applied for post hoc pairwise comparisons. Associations between categorical variables were assessed using the chi-square test or Fisher’s exact test, as appropriate.

Descriptive statistics were reported as mean ± standard deviation for continuous variables, and as frequencies and percentages for categorical variables. Data visualization was performed using bar charts with error bars representing mean ± standard deviation (IBM corp 2011).

### In silico studies

#### Docking studies

For the in-silico studies, molecules 4 and 6 were selected based on their IC₅₀ values, which identified them as the most potent molecules. Following the gene expression analysis, the target protein to be used for molecular docking was subsequently selected.

The molecular docking studies were performed using AutoDock 4.2 to explore the binding interactions of molecules** 4** and **6** with the protein structures of Caspase-8, ATG8, AMBRA1, and BAX. The 3D structures of these target proteins were obtained from the RCSB Protein Data Bank (Berman et al. 2003). The chemical structures of molecules** 4** and **6** were generated using GaussView and subsequently optimized through Density Functional Theory (DFT) calculations employing the B3LYP functional and the 6–31G basis set within the Gaussian software package (Frisch et al. 2009). Prior to docking, protein structures were prepared by removing crystallographic ligands and water molecules, followed by the addition of polar hydrogen atoms and partial atomic charges to ensure accurate representation of molecular interactions.

#### MD simulation

In this work, we have performed molecular dynamics (MD) simulations of the complex structures from docking using GROMACS 2025.4 (Abraham et al. 2025) with force field charm 36-jul2022 (Yu et al. 2021).

To prepare the system for MD simulation, the uncomplexed protein or the protein–ligand complex was placed at the center of a cubic box with periodic boundary conditions, and thereafter, the system was solvated by adding water with the code TIP3 and neutralized. Next, the system was energy-minimized using the steepest descent algorithm. Then, the solvated and neutralized system was subjected to NVT and NPT simulation of 1 ns for each, then the MD stimulation for 50 ns (Vivek-Ananth et al. 2022). With GROMACS algorithm, RMSD, RMSF and H-bond were calculated (Gorelov et al. 2024).

### *In ovo* hen’s egg test on chorioallantoic membrane (HET-CAM)

The hen’s egg chorioallantoic membrane (HET-CAM) assay was performed according to the Interagency Coordinating Committee on the Validation of Alternative Methods (ICCVAM) evaluation criteria for alternative ocular and dermal irritation methods (ICCVAM 2010; Luepke 1985). According to ICCVAM guidelines, the HET-CAM test conducted before embryonic day 14 is not classified as an animal experiment; therefore, no ethics committee approval is required. Fertilized eggs (*n* = 3) were incubated until day 7 at 37.5 °C and 55–65% humidity. After windowing (2 × 2 cm) at the equator of the eggshell on day 7, 300 μL of each test molecule (4 and 6) was directly applied onto the CAM surface. Following application, the CAM was examined by direct visual inspection, and the onset times of hemorrhage (tH), vascular lysis (tL) and coagulation (tC) were recorded over a 300-s observation period. As specified in ICCVAM guidance, if no reaction occurred within this interval, the corresponding endpoint was assigned a value of 301 s. In accordance with the reference method, 0.9% NaCl (sodium chloride) was used as the negative control, while 0.1 M NaOH (sodium hydroxide) served as the positive control.

The irritation severity score (IS) was calculated as follows:

$$IS=5^\ast\left(\left(301-tH\right)/300\right)+7^\ast\left(\left(301-tL\right)/300\right)+9^\ast\left(\left(301-tC\right)/300\right)$$

The Irritation Score (IS) was calculated using the ICCVAM-recommended weighted equation (maximum possible value: 21). In this method, tH, tL, and tC represent the time (in seconds) to the first appearance of hemorrhage, vascular lysis, and coagulation, respectively. Based on the IS value, samples were classified as non-irritant (0–0.9), slight irritant (1.0–4.9), moderate irritant (5.0–8.9), or severe irritant (≥ 9).

## Supplementary information

Below is the link to the electronic supplementary material.

Supplementary file1 (DOCX 2375 KB)

## Data Availability

All figures and schemes presented in this article are original and were generated by the authors during the course of the study. The supporting spectral data (including NMR spectra) for all synthesized molecules are available in the Supplementary Information file submitted with this article. No external datasets, software, or code were used or generated in this study.
