# Supplementary material for: Design, synthesis, and mechanistic evaluation of propargylated salicylaldehyde derivatives as dual apoptosis–autophagy modulator for pancreatic cancer
Source: Naunyn Schmiedebergs Arch Pharmacol. 2026 Feb 24;399(8):11785–804. doi: 10.1007/s00210-026-05127-w (PMC13269460; doi:10.1007/s00210-026-05127-w)
Supplement: Supplementary file 1 — Supplementary file1 (DOCX 2375 KB) [file 210_2026_5127_MOESM1_ESM.docx]

**Design, Synthesis, and Mechanistic Evaluation of Propargylated Salicylaldehyde Derivatives as Dual Apoptosis–Autophagy Modulator for Pancreatic Cancer**

Filiz Taspinar,^a^ * Karina Amudi,^b^ Nurettin Menges^b,c^ *

^a^ Department of Physiology, Faculty of Medicine, Aksaray University, 68300, Aksaray, Türkiye

^b^ Science and Technology Research and Application Center (BITAM), Necmettin Erbakan University, 42090 Konya, Türkiye

^c^ Biomedical Engineering, Faculty of Engineering, Necmettin Erbakan University, 42090, Konya, Türkiye

* Corresponding authors: [filiztaspinar@aksaray.edu.tr](mailto:filiztaspinar@aksaray.edu.tr); [nurettin.menges@erbakan.edu.tr](mailto:nurettin.menges@erbakan.edu.tr)

**Table of Content Pages**

Copy of NMR Spectra S2

Copy of Melting Curve and Amplification Spectra for Studied genes S12

HRMS spectra for unknown molecules S19

Figure S42: Dose responses of DMSO in cell culture S20

MD simulation graphs for molecule 4 and 6 S21

**Copy of NMR spectra for all molecules**.


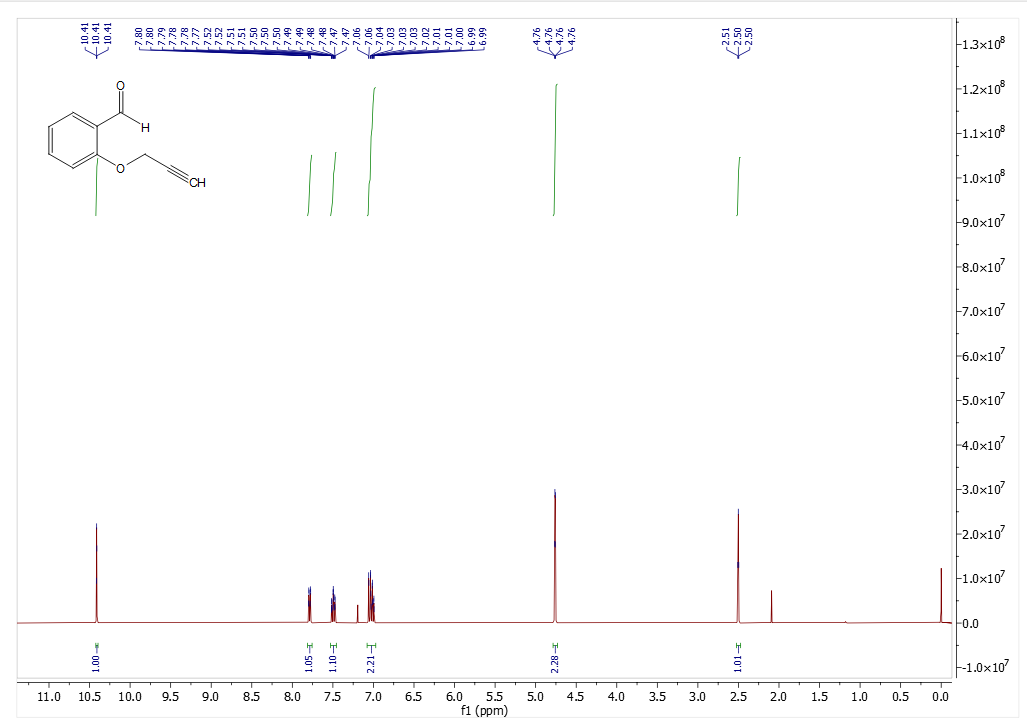


**Figure S1 1H-NMR Spectra of compound 1**

**
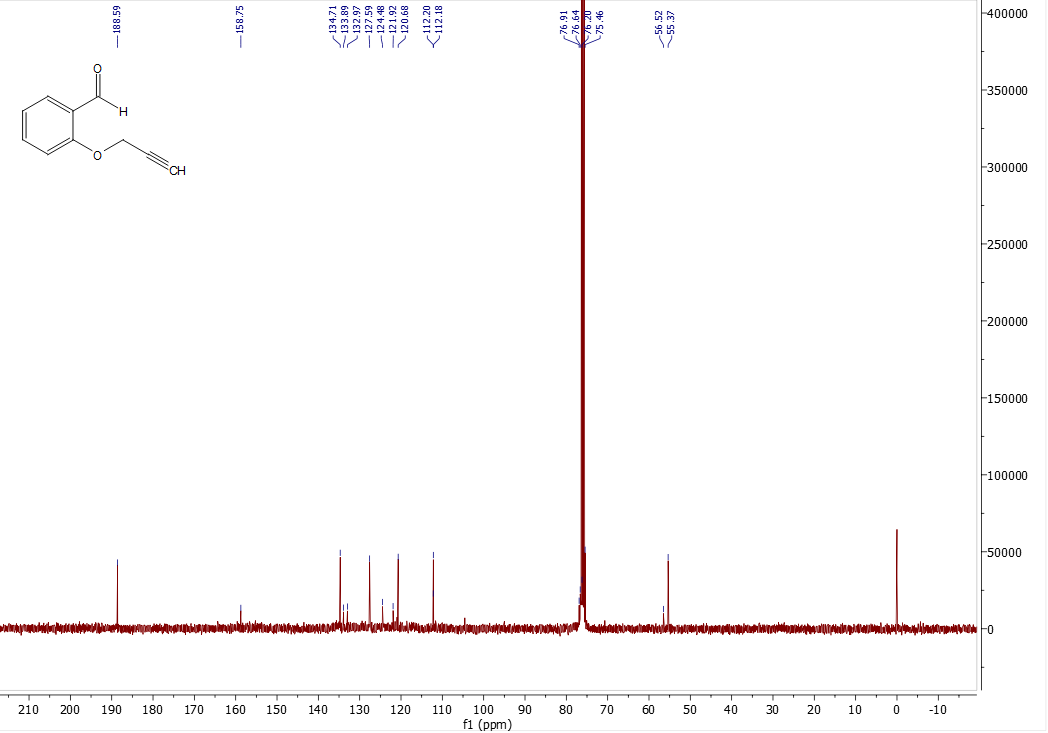
**

**Figure S2 13C-NMR Spectra of compound 1**


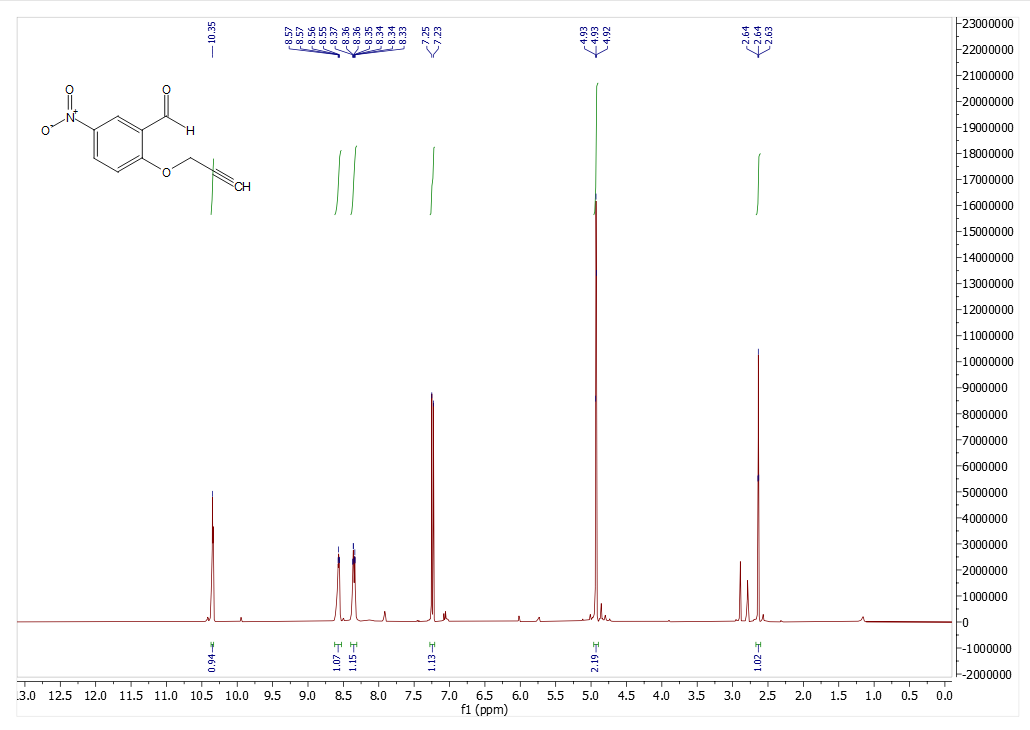


**Figure S3 1H-NMR Spectra of compound 2**


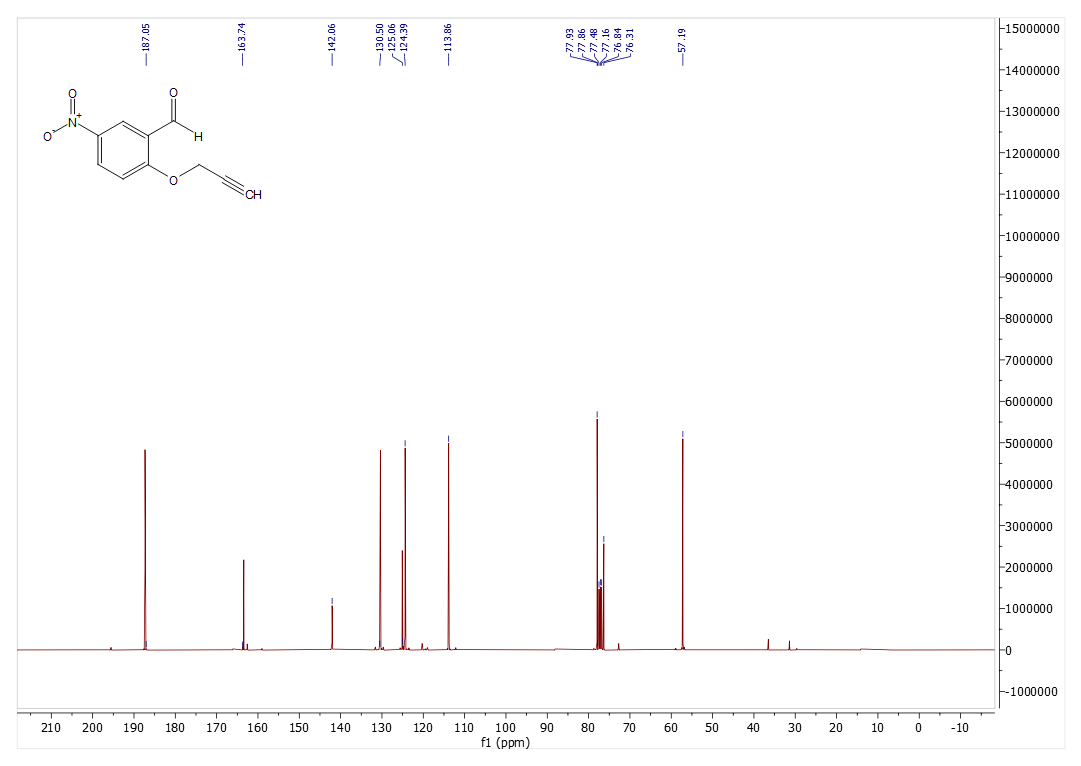


**Figure S4 13C-NMR Spectra of compound 2**


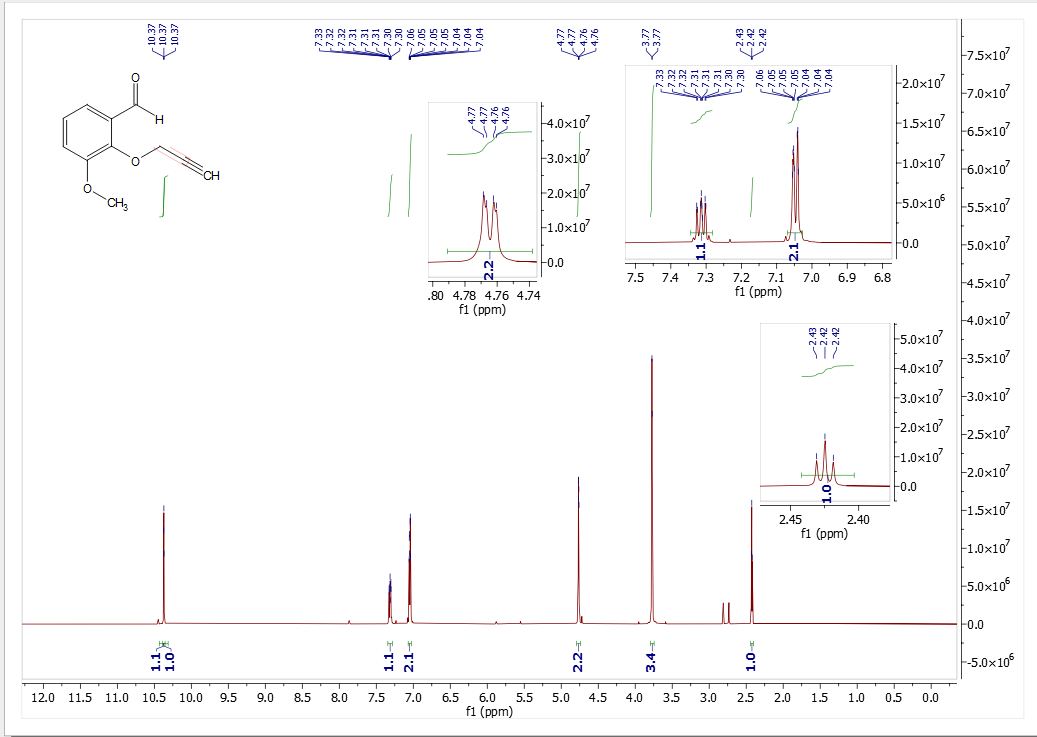


**Figure S5 1H-NMR Spectra of compound 3**


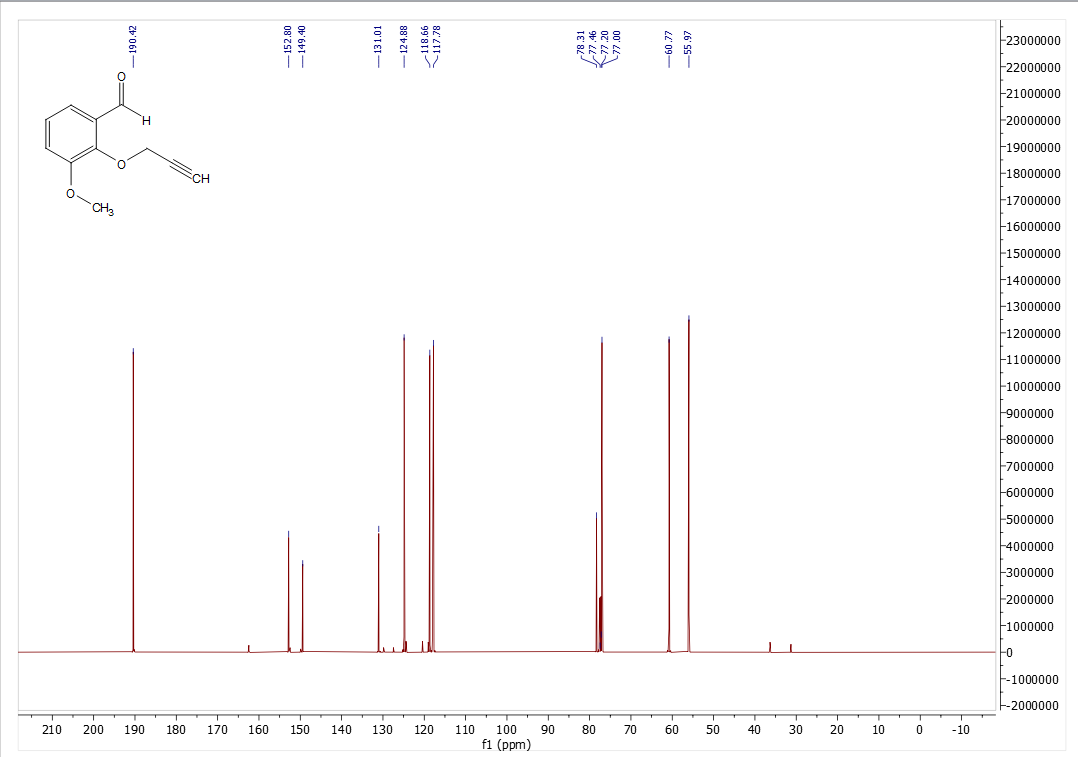


**Figure S6 13C-NMR Spectra of compound 3**


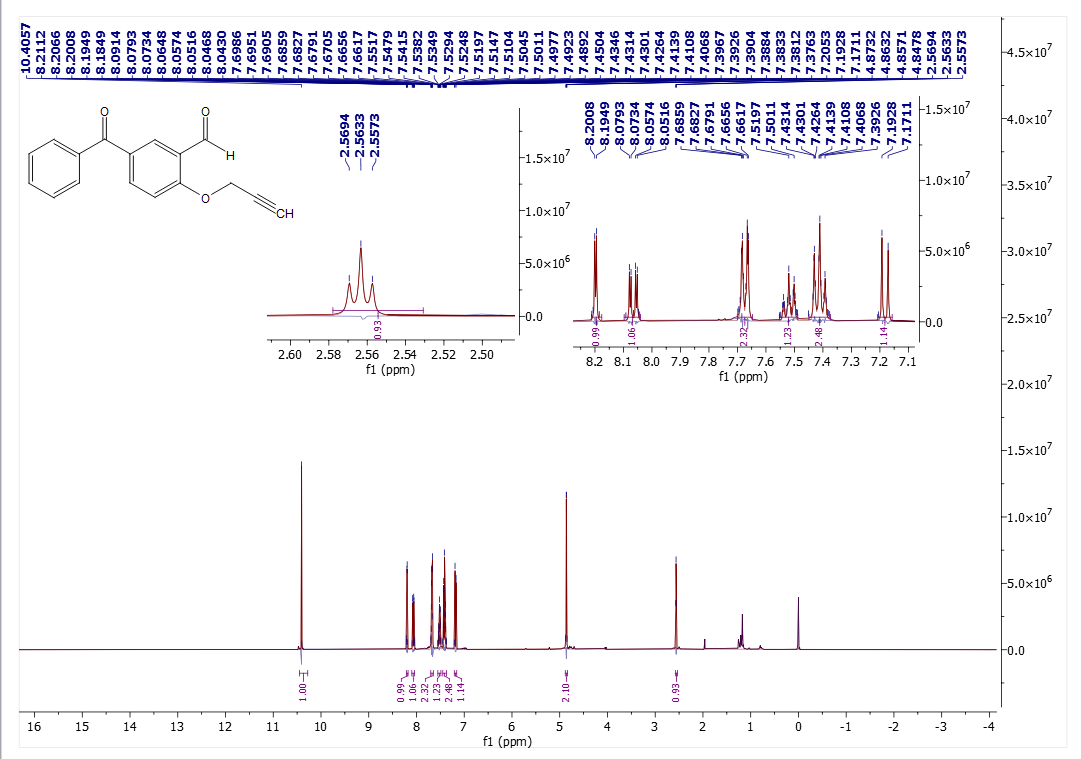


**Figure S7 1H-NMR Spectra of compound 4**


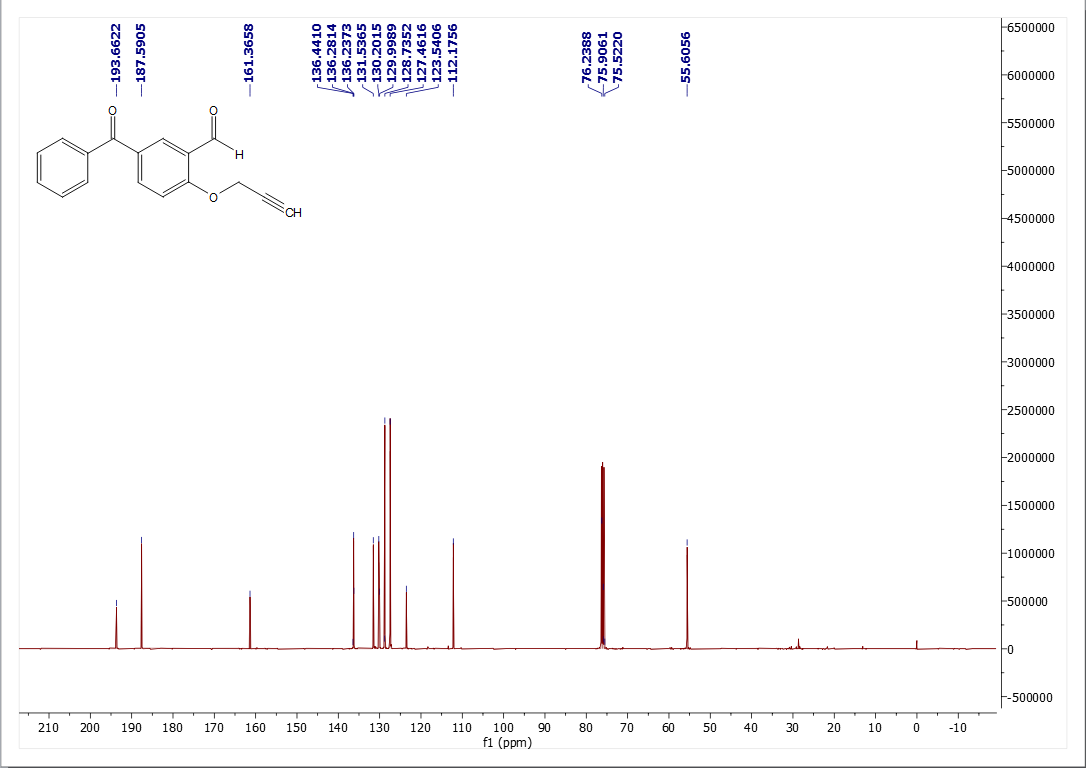


**Figure S8 13C-NMR Spectra of compound 4**


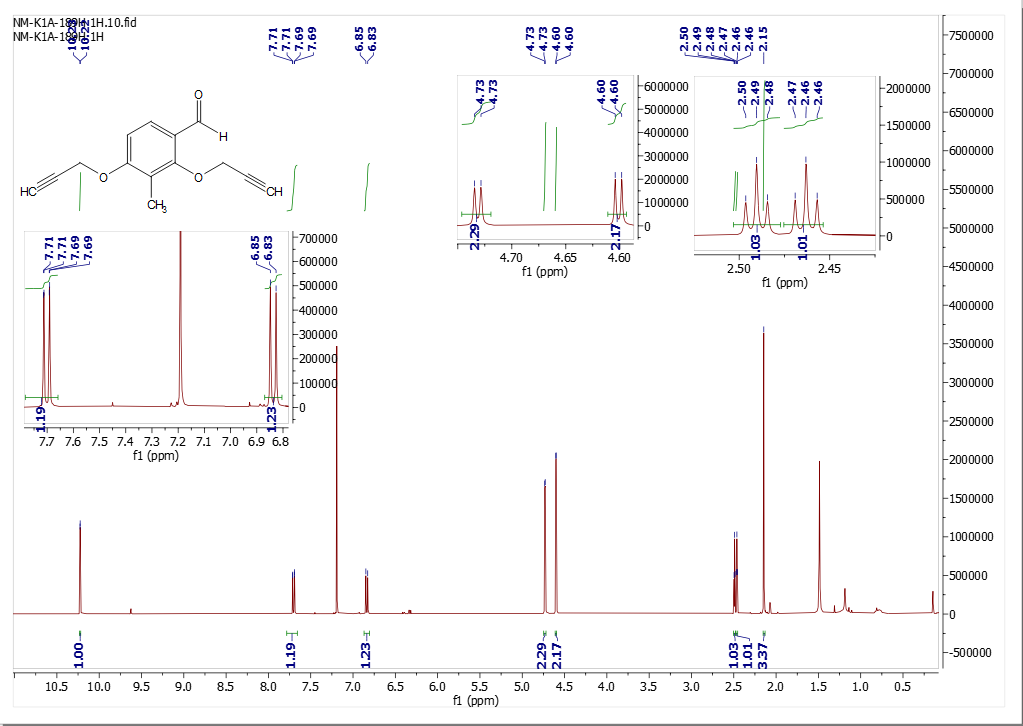


**Figure S91H-NMR Spectra of compound 5**


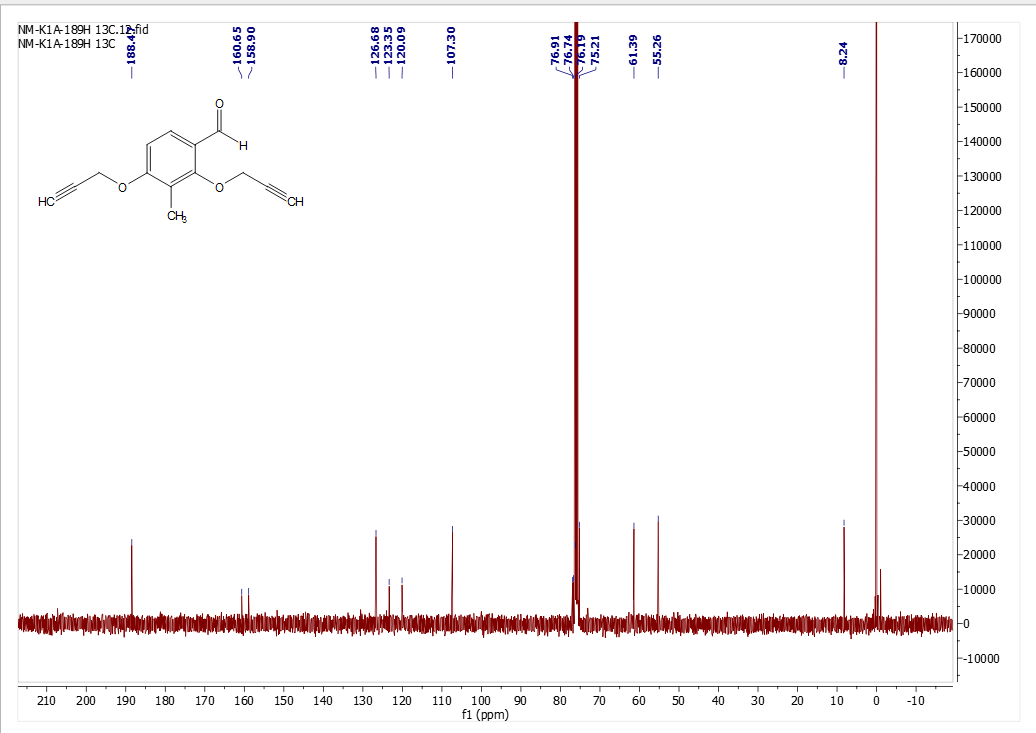


**Figure S10 13C-NMR Spectra of compound 5**


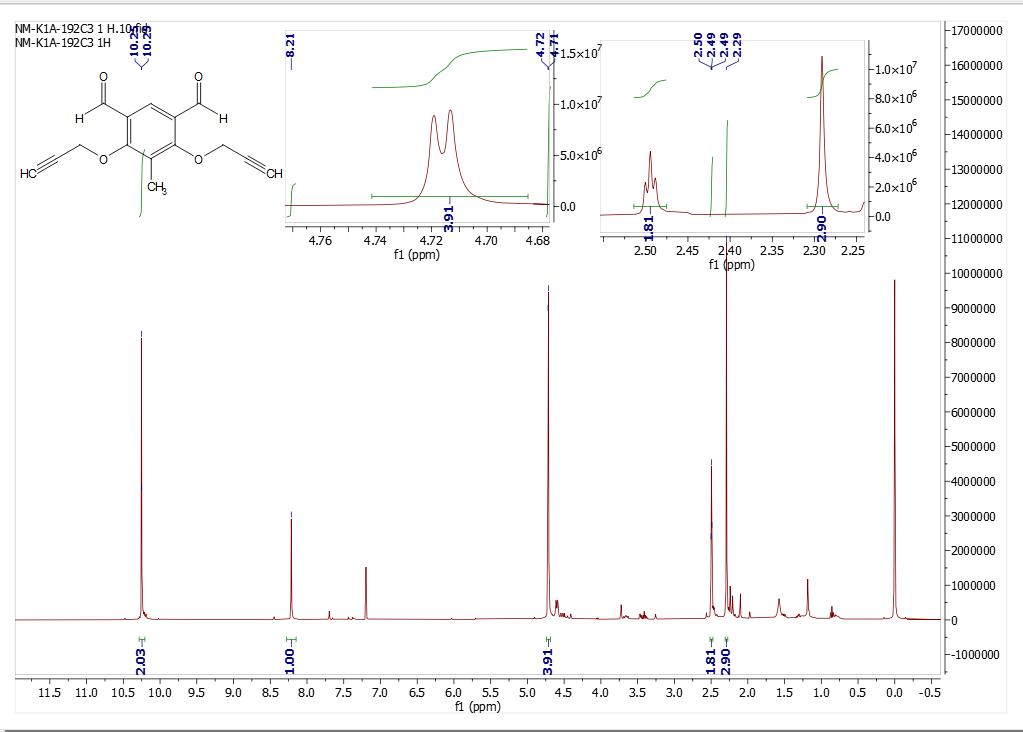


**Figure S11 1H-NMR Spectra of compound 6**


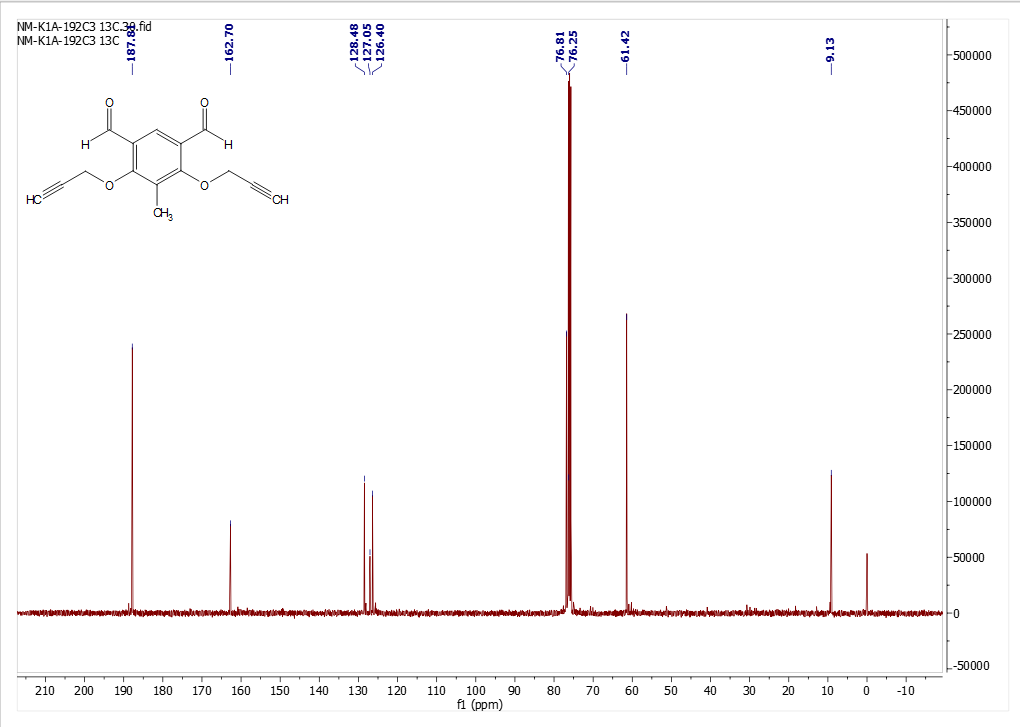


**Figure S12 13C-NMR Spectra of compound 6**


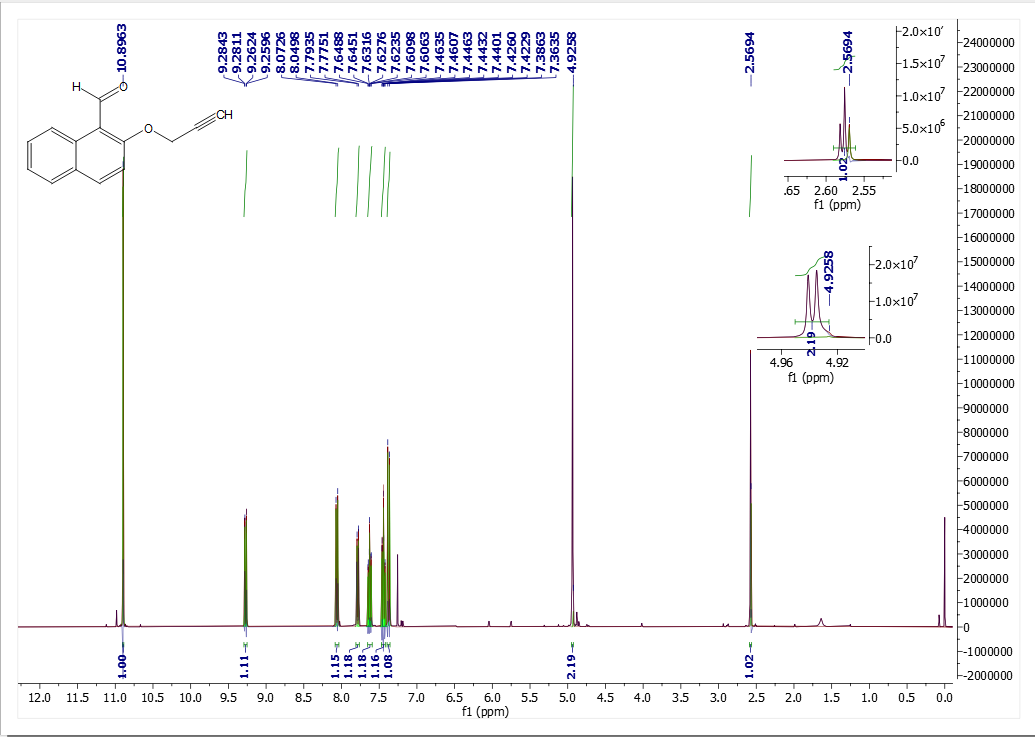


**Figure S13 1H-NMR Spectra of compound 7**


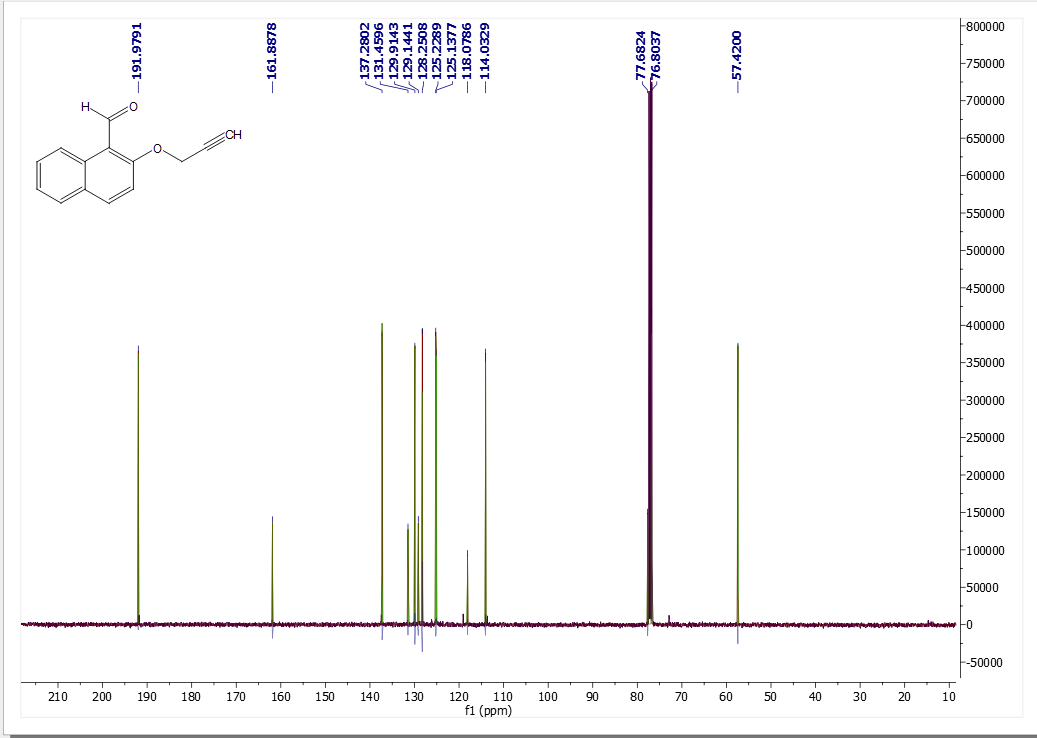


**Figure S14 13C-NMR Spectra of compound 7**


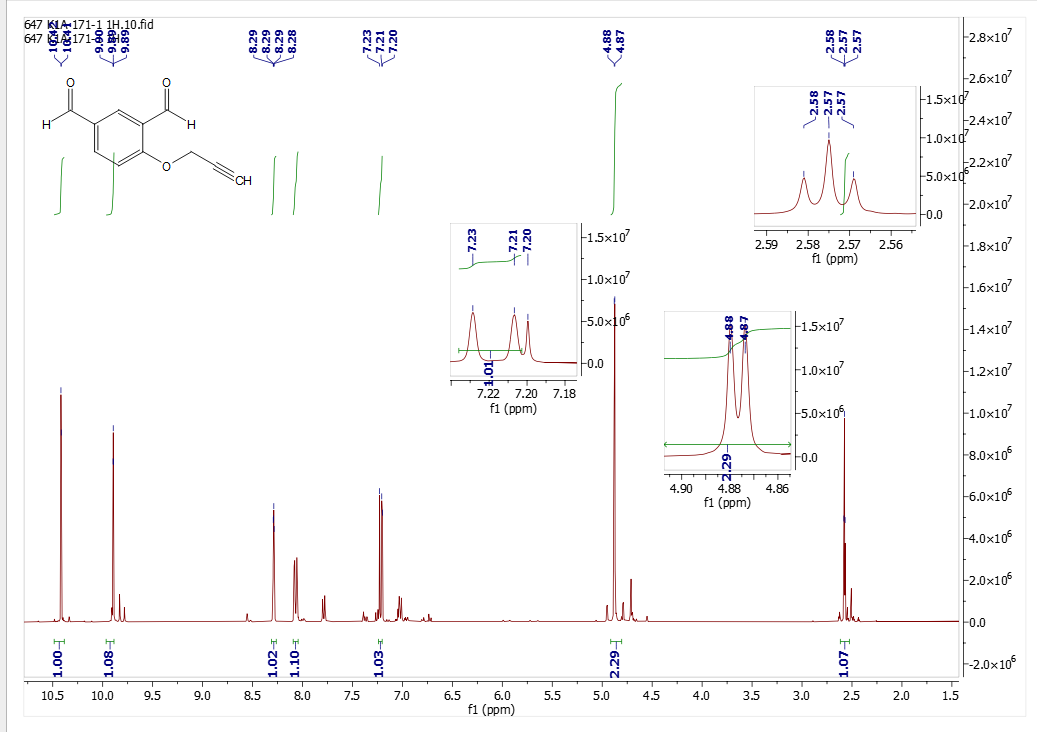


**Figure S15 1H-NMR Spectra of compound 8**


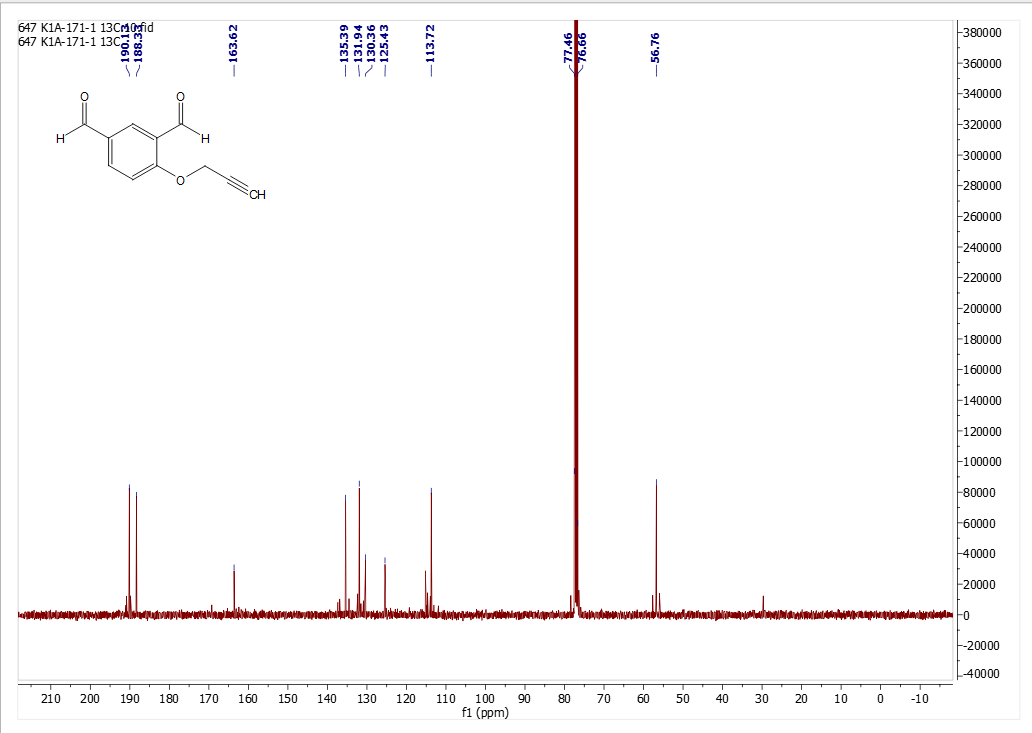


**Figure S16 13C-NMR Spectra of compound 8**


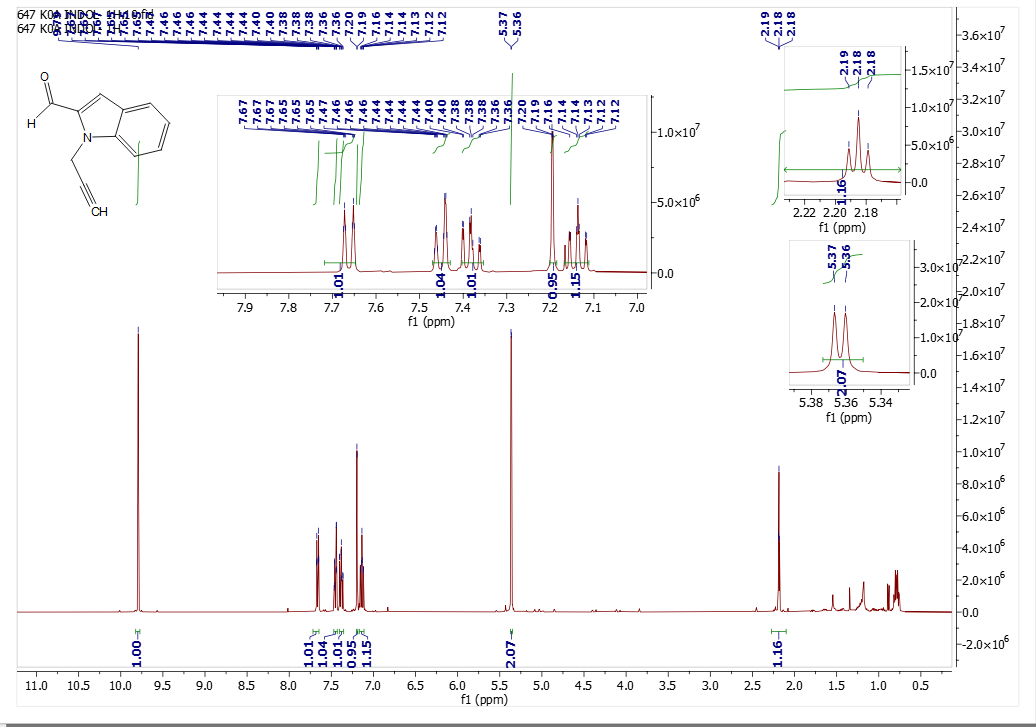


**Figure S17 1H-NMR Spectra of compound 9**


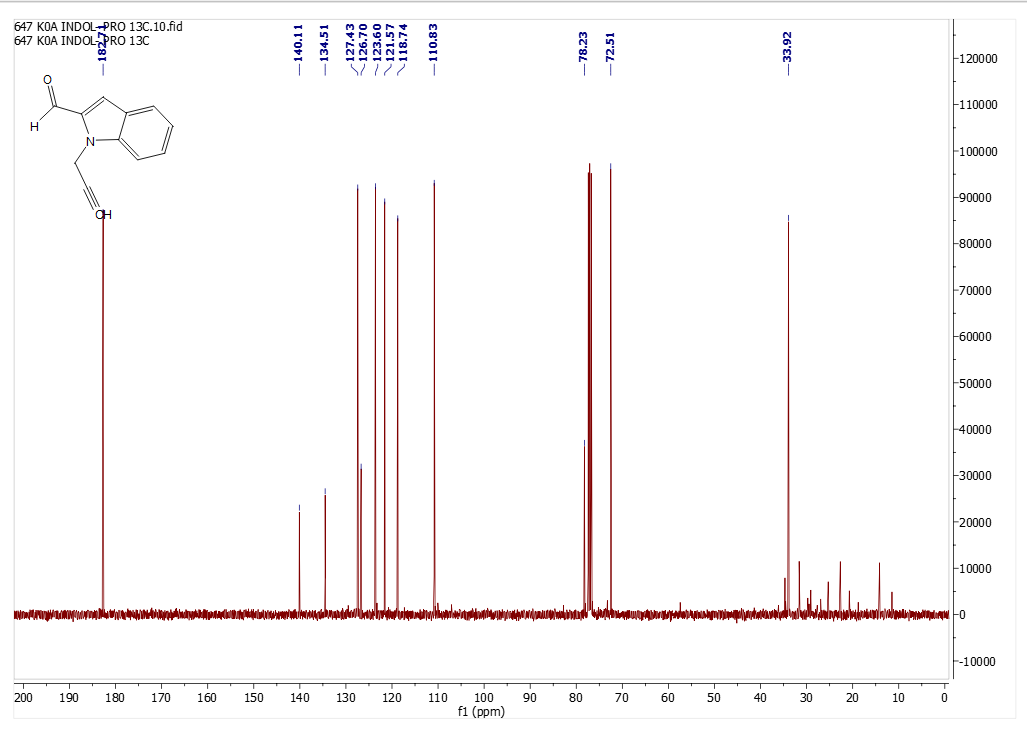


**Figure S18 13C-NMR Spectra of compound 9**


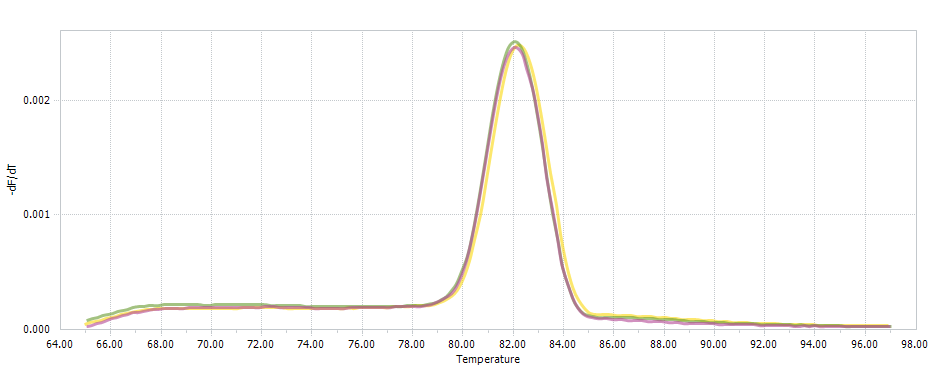
**Figure S19. Melting Curve for ATG 8**


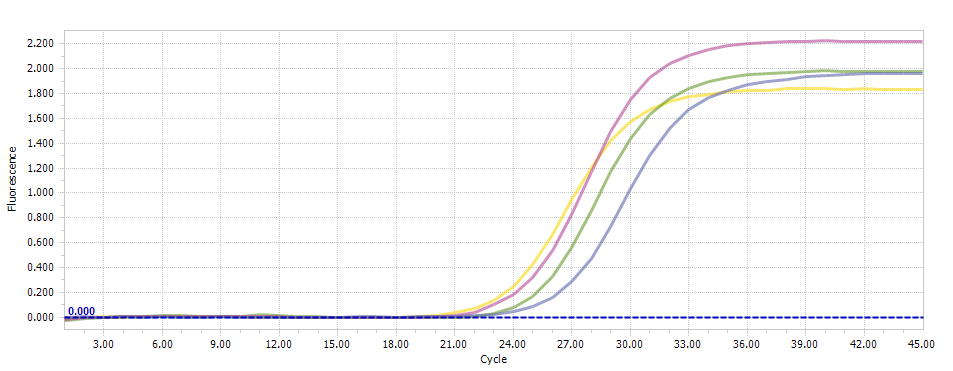


**Figure S20. Amplification Plot for ATG 8**


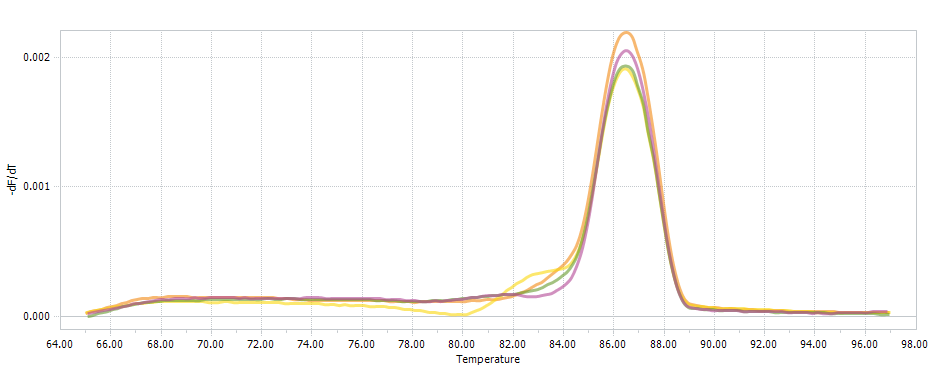


**Figure S21. Melting Curve for ATG 6**


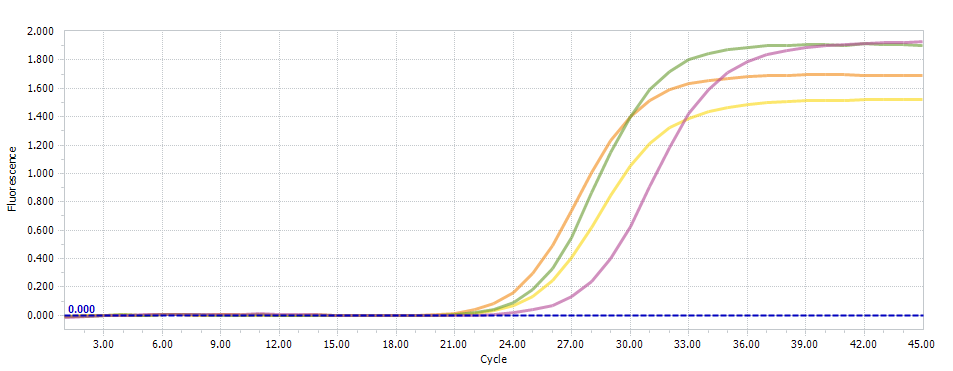


**Figure S22. Amplification Plot for ATG 6**


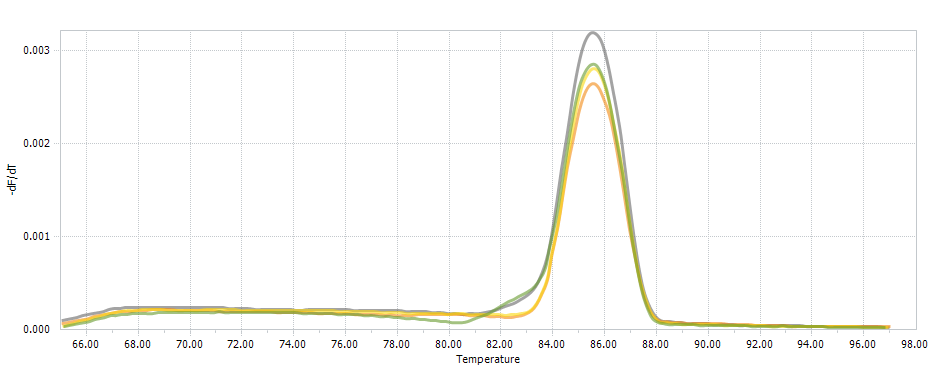
**Figure S23. Melting Curve for CASP 9**
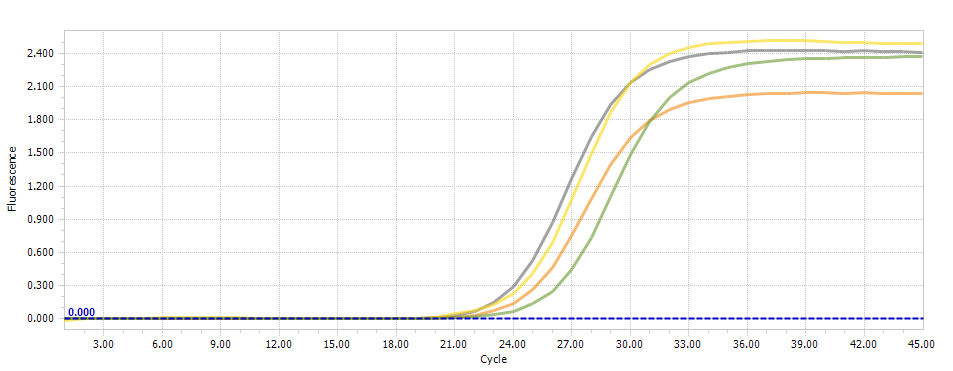


**Figure S24. Amplification Plot for CASP 9**


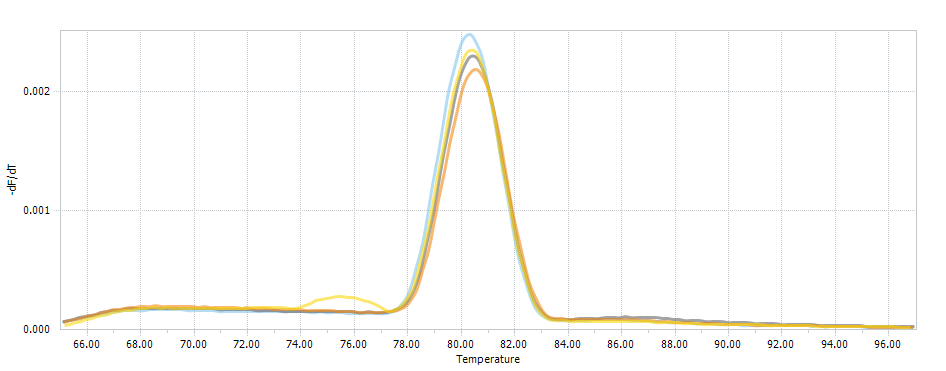


**Figure S25. Melting Curve for CASP 8**


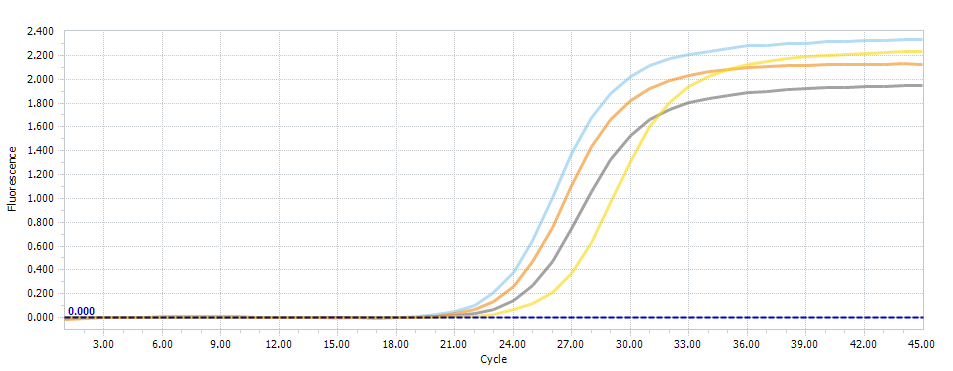


**Figure S26. Amplification Plot for CASP 8**


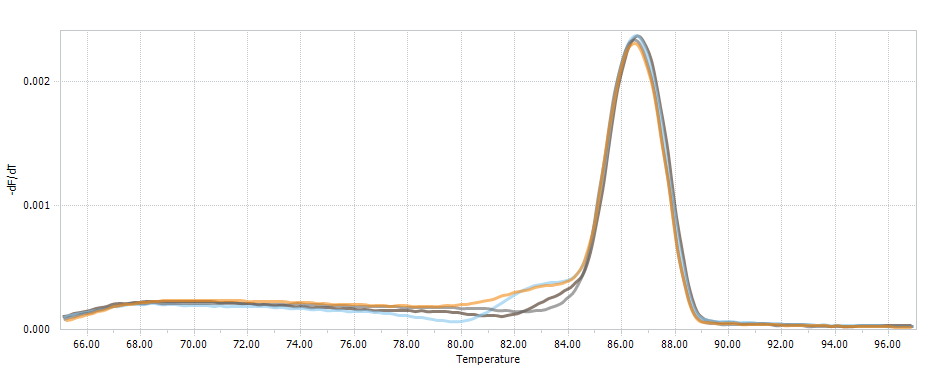


**Figure S27. Melting Curve for BCX_L_**
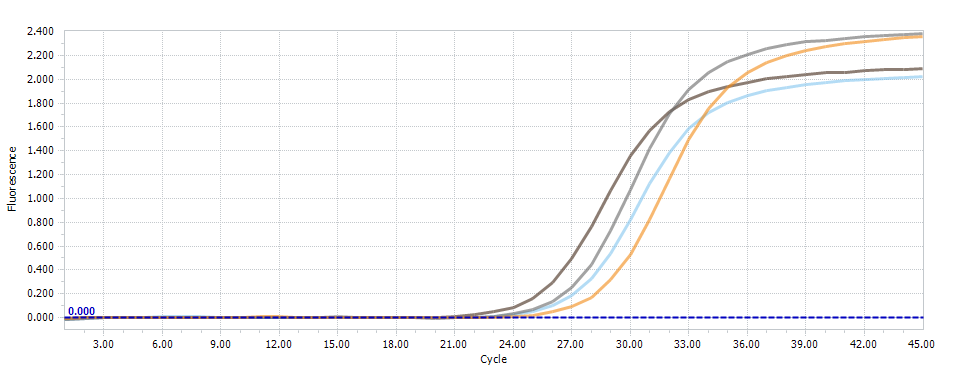


**Figure S28. Amplification Plot for BCX_L_**


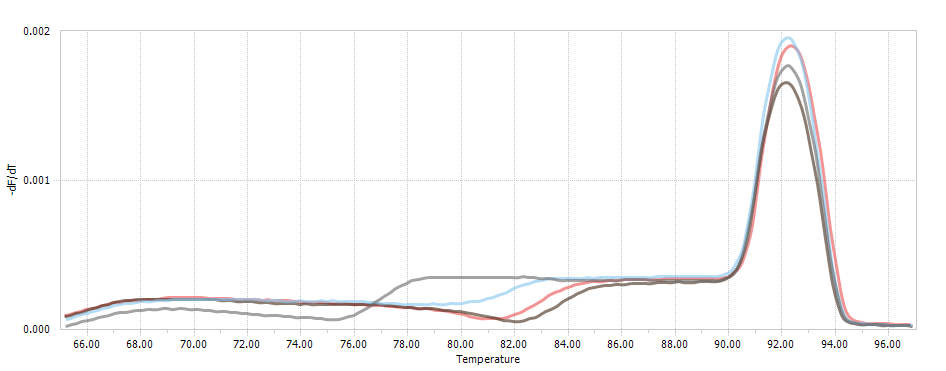


**Figure S29. Melting Curve for BAD**


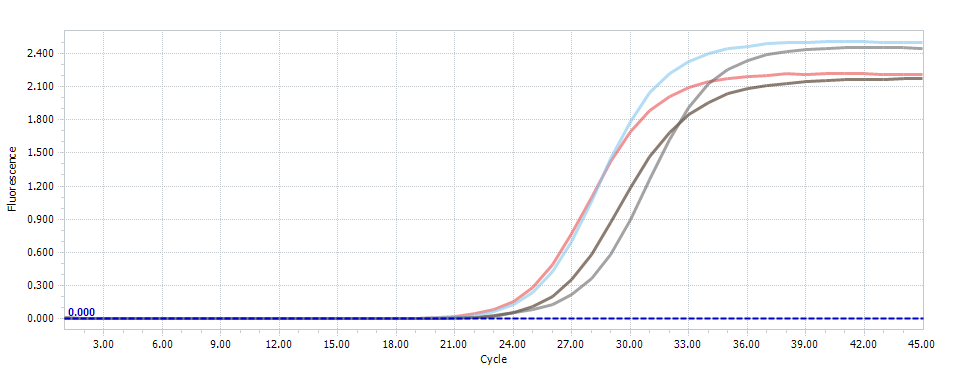


**Figure S30. Amplification Plot for BAD**


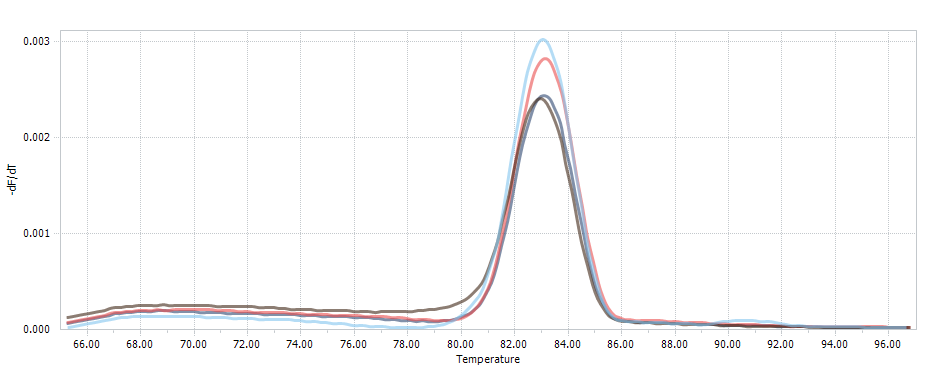


**Figure S31. Melting Curve for GAPDH**


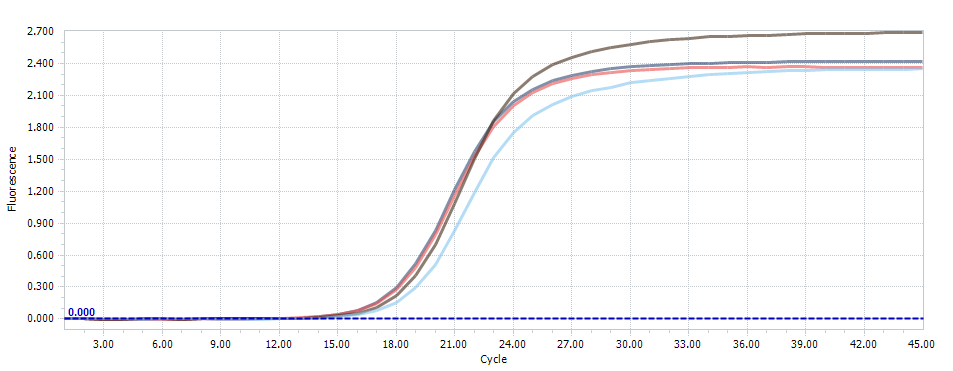


**Figure S32. Amplification Plot for GAPDH**


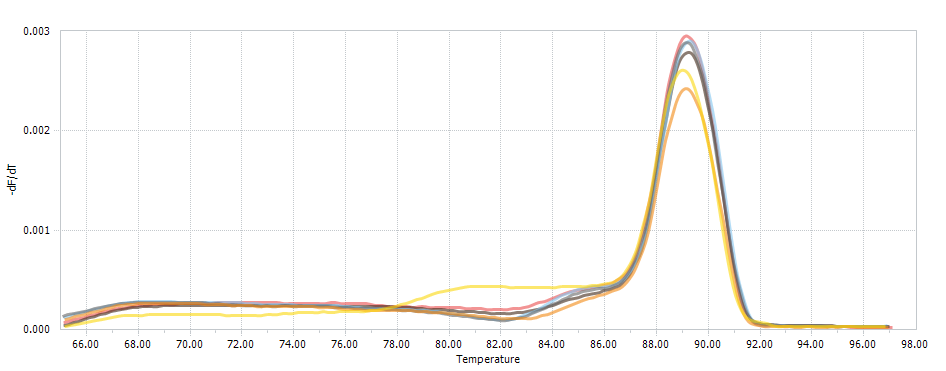


**Figure S33. Melting Curve for BAX**


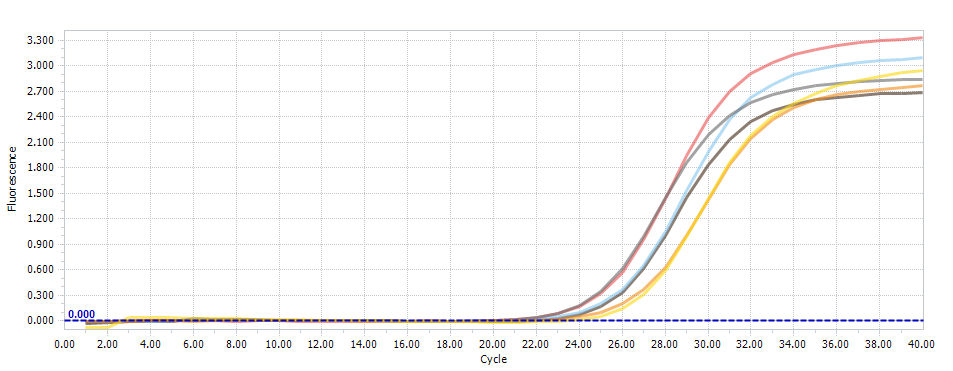


**Figure S34. Amplification Plot for BAX**


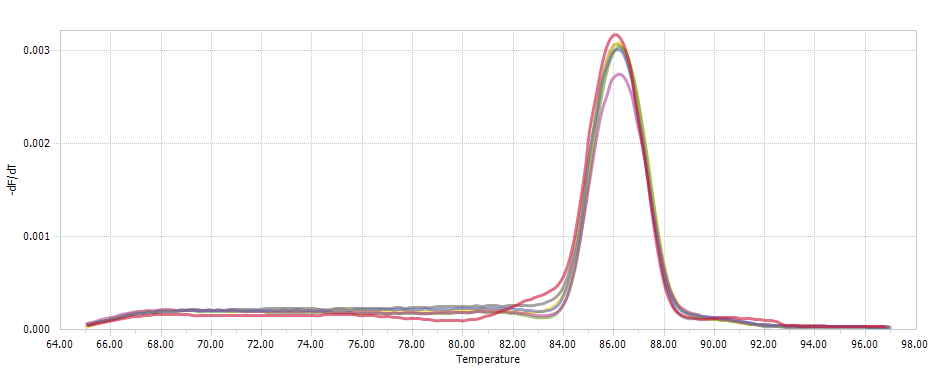


**Figure S35. Melting Curve for AMBRA1**
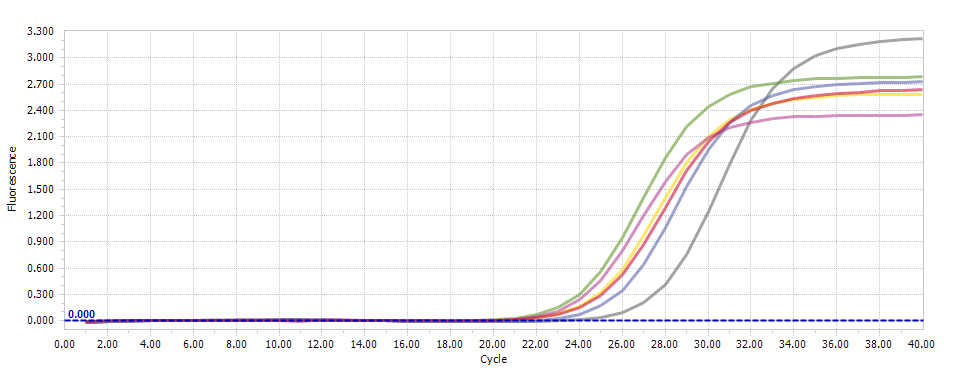


**Figure S36. Amplification Plot for AMBRA**


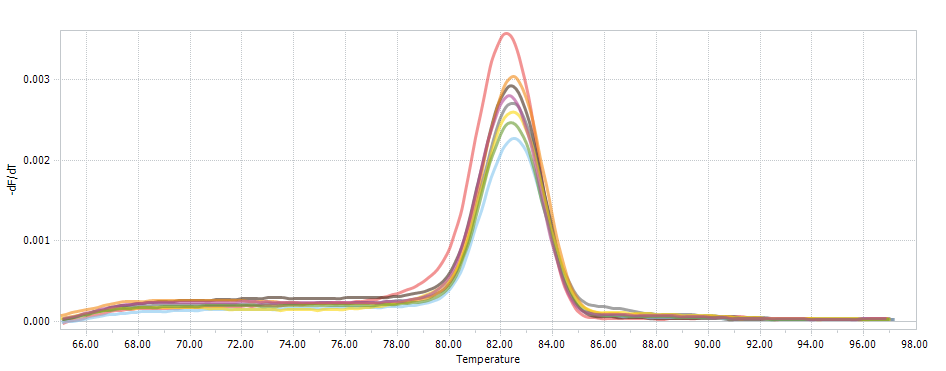


**Figure S37. Melting Curve for ATG 7**


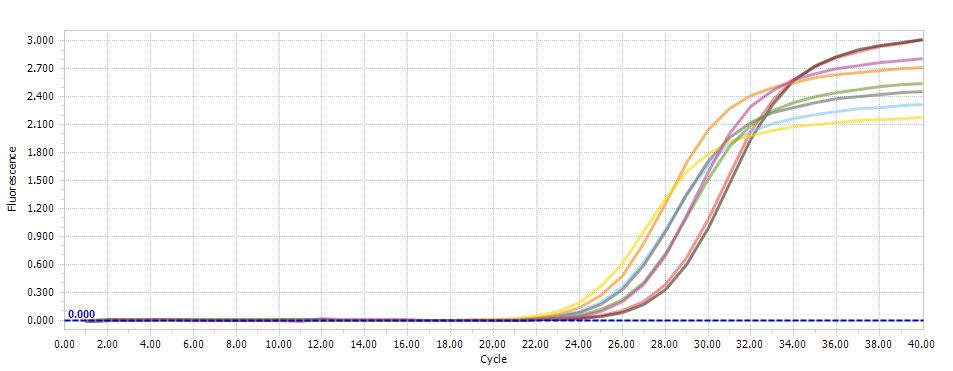


**Figure S38. Amplification Plot for ATG 7**

**
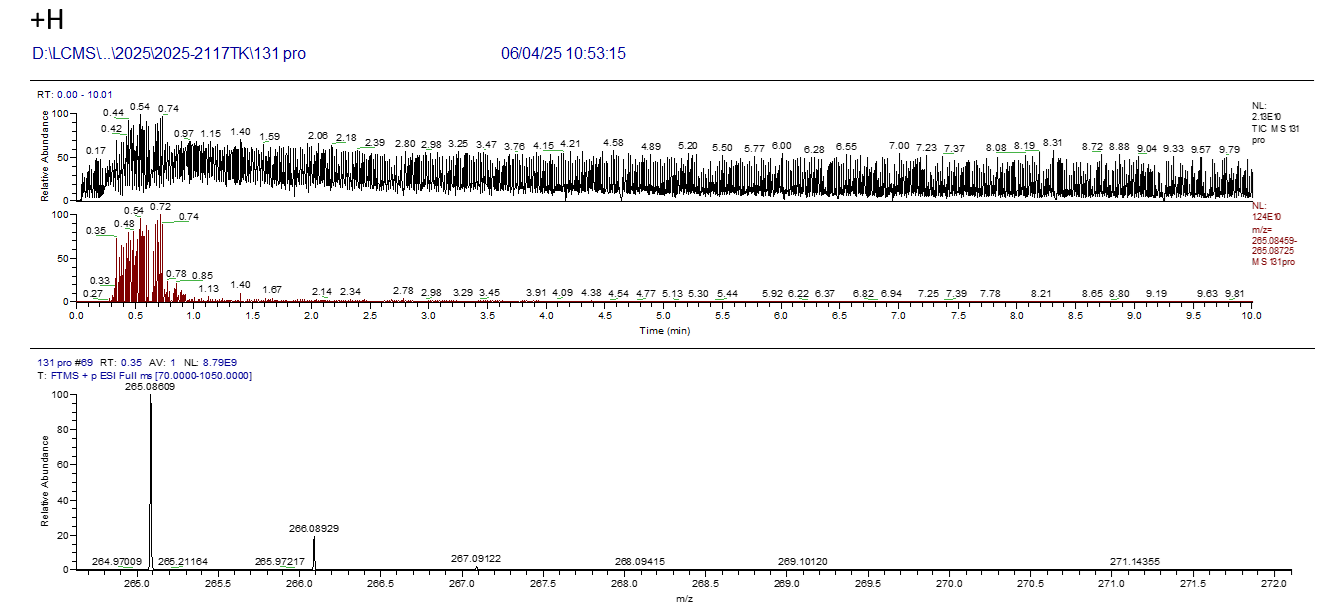
**

**Figure S39. HRMS spectrum of compound 4**

**
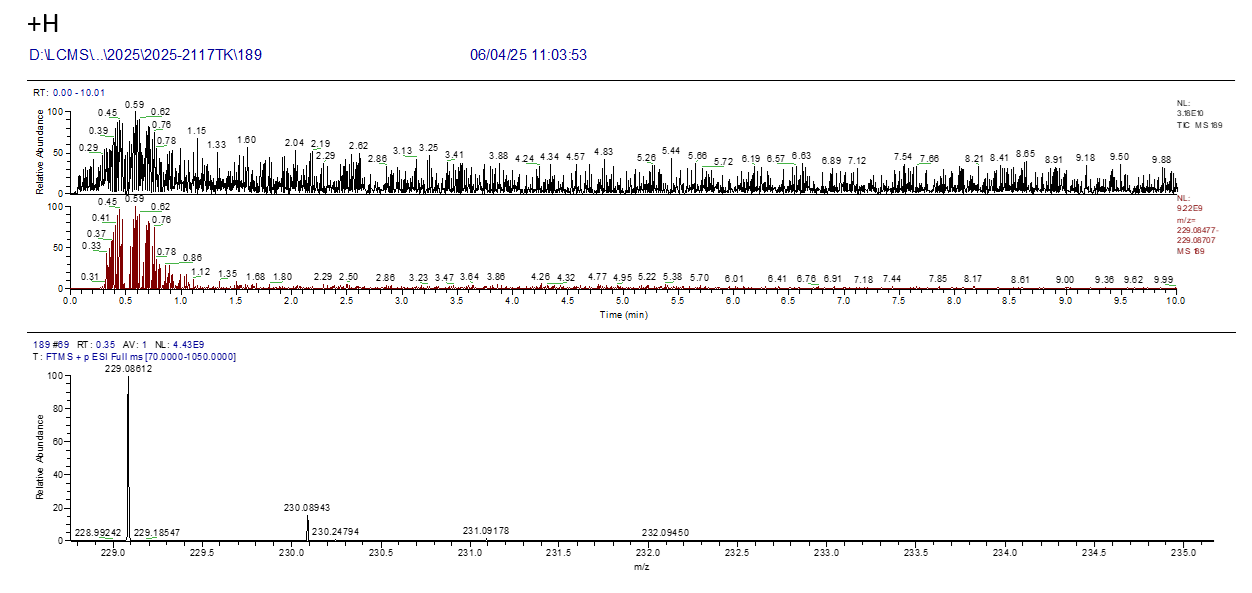
**

**Figure S40. HRMS spectrum of compound 5**

**
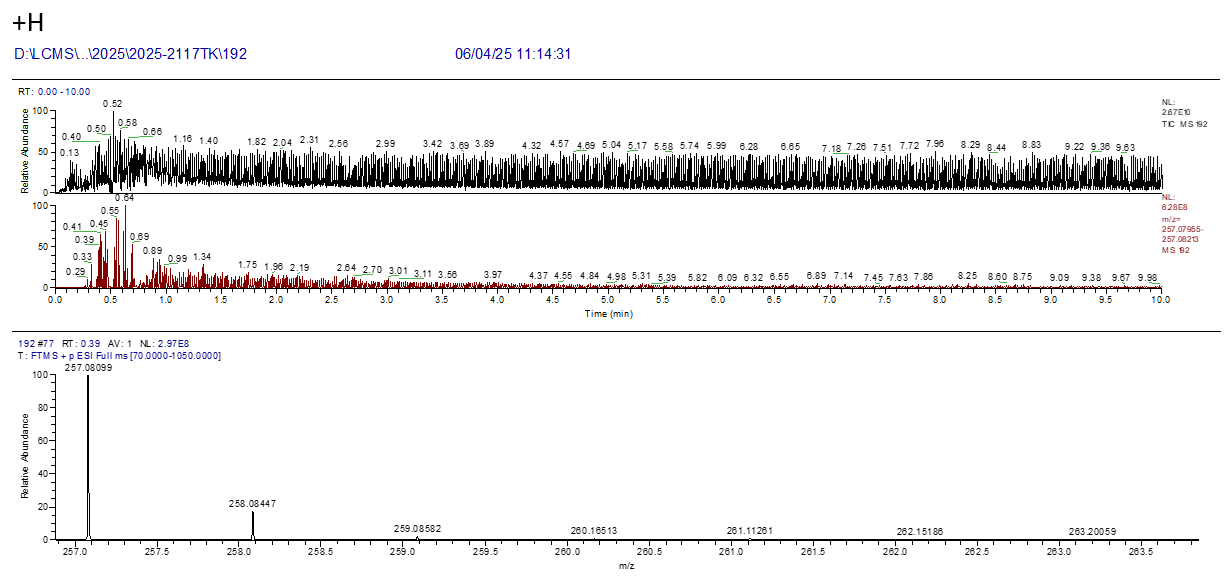
**

**Figure S41. HRMS spectrum of compound 6**

**Figure S42. Dose responses of DMSO in cell culture.**


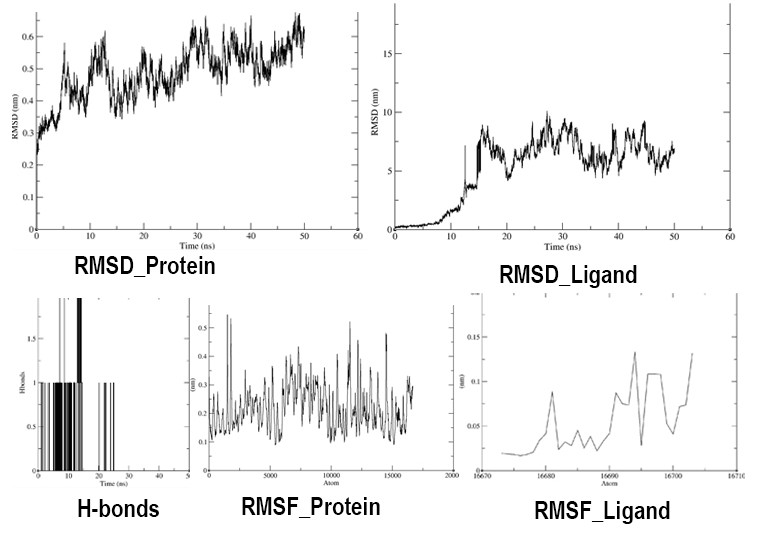


**Figure S43. Protein-Ligand MD simulation for molecule 6 with 8WQR protein**


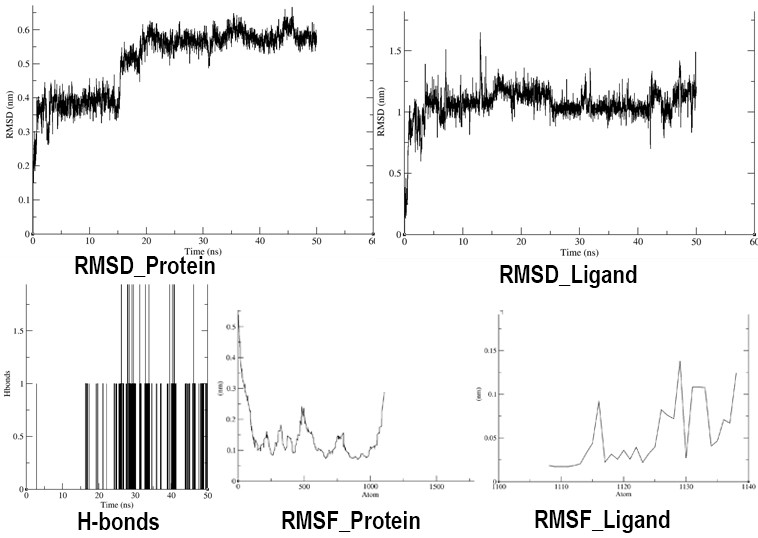


**Figure S44. Protein-Ligand MD simulation for molecule 6 with 8G1T protein**


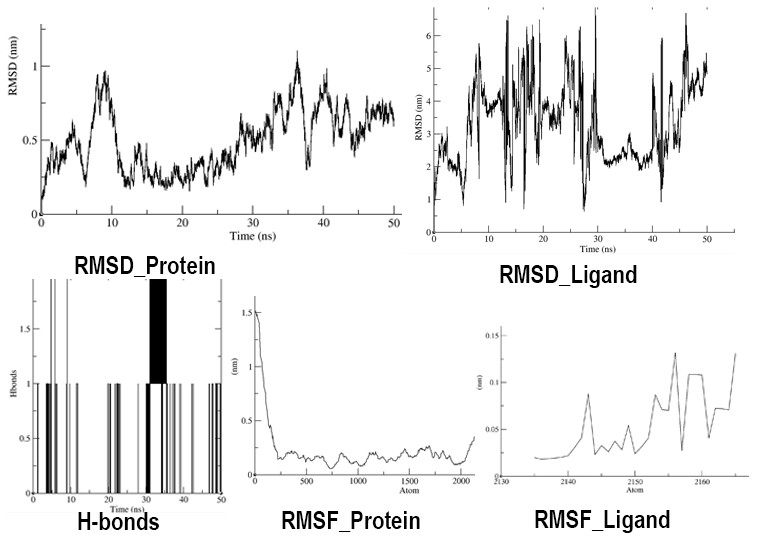


**Figure S45. Protein-Ligand MD simulation for molecule 6 with 6HB9 protein**


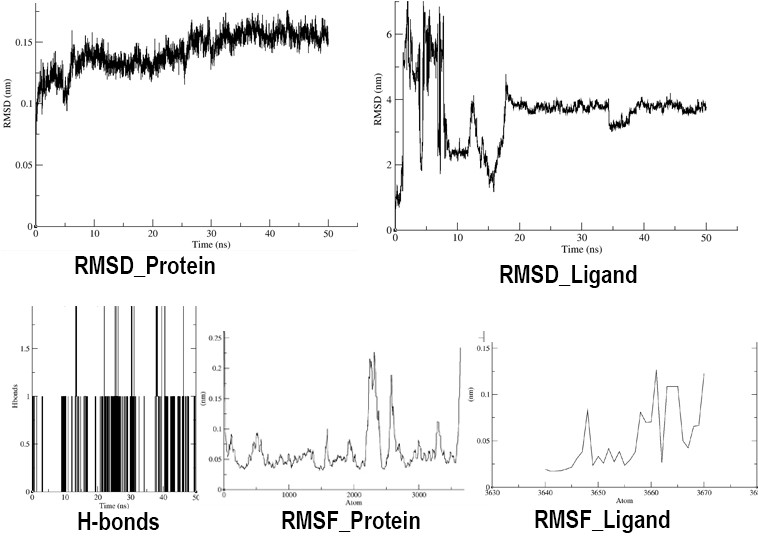


**Figure S46. Protein-Ligand MD simulation for molecule 6 with 6H11 protein**


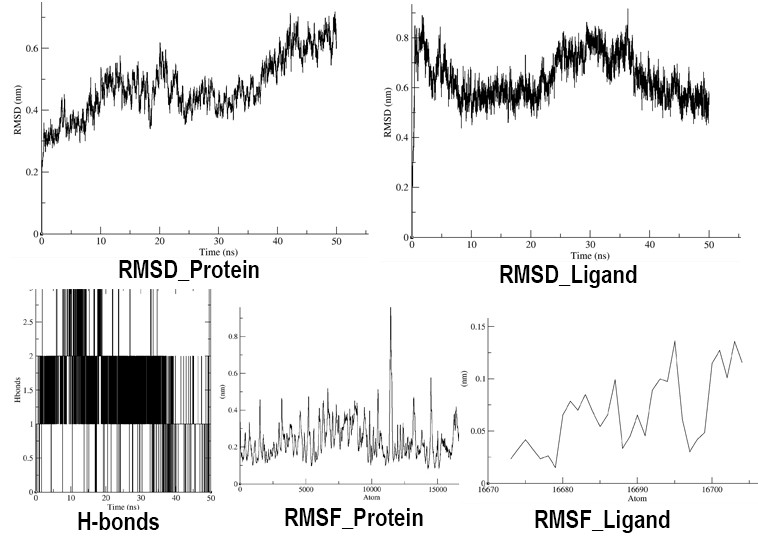


**Figure S47. Protein-Ligand MD simulation for molecule 4 with 8WQR protein**


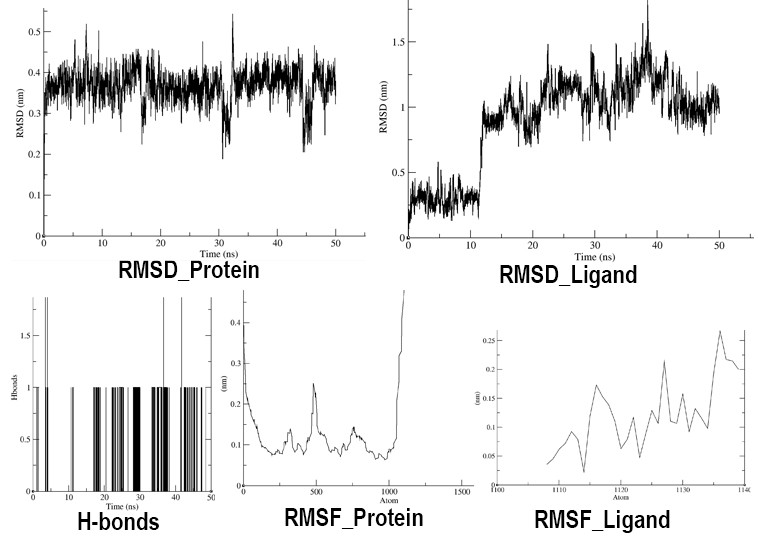


**Figure S48. Protein-Ligand MD simulation for molecule 4 with 8G1T protein**


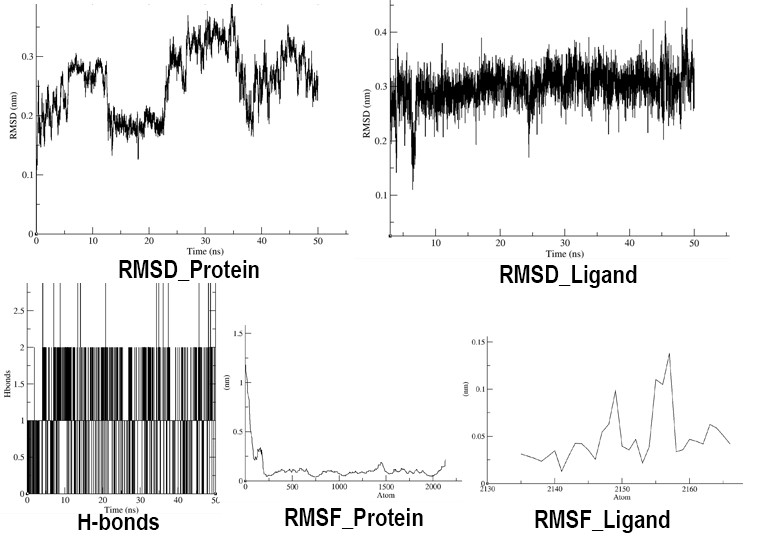


**Figure S49. Protein-Ligand MD simulation for molecule 4 with 6HB9 protein**


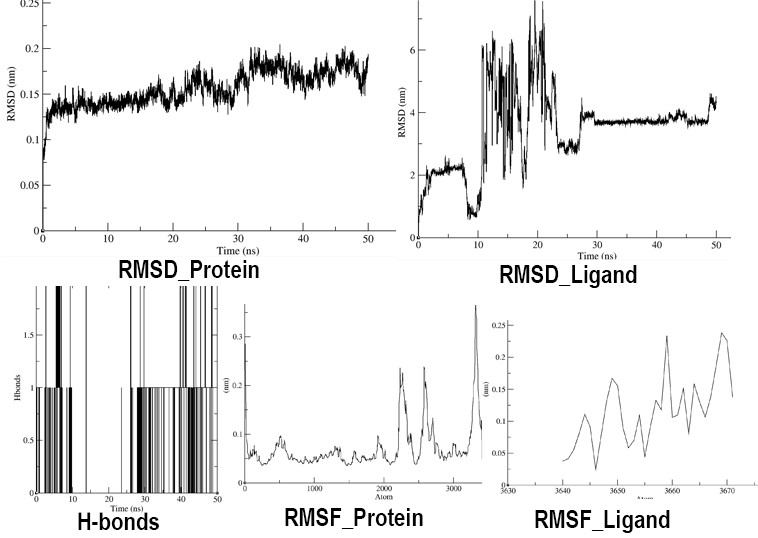


**Figure S50. Protein-Ligand MD simulation for molecule 4 with 3H11 protein**
